# ACTN2, regulated by PRDM9, affects the growth and inflammation of vascular smooth muscle cells by interacting with PDLIM1 in intracranial aneurysms

**DOI:** 10.3389/fnmol.2025.1606973

**Published:** 2025-08-13

**Authors:** Guangxu Zhang, Jinbing Zhao, Zhiqiang Yu, Hongyi Liu

**Affiliations:** Department of Neurosurgery, Affiliated Nanjing Brain Hospital, Nanjing Medical University, Nanjing, China

**Keywords:** ACTN2, PDLIM1, PRDM9, H3K4me3, intracranial aneurysms, VSMCs

## Abstract

**Background:**

The occurrence of aneurysms is closely related to the growth and inflammatory response of vascular smooth muscle cells (VSMCs). The regulatory mechanism of ACTN2 in intracranial aneurysms (IA) has not yet been fully elucidated. This study aims to reveal the role of the PRDM9-ACTN2-PDLIM1 axis in the progression of aneurysms and its impact on VSMCs.

**Methods:**

By integrating GEO datasets (GSE54083, GSE75436) and protein-protein interaction network analysis, ACTN2 was identified as a key gene. Techniques such as shRNA/overexpression, tissue staining, immunofluorescence, ELISA, and Western blot were used to analyze the effects of ACTN2 on VSMC proliferation, apoptosis, inflammation, and the Hippo pathway. The transcriptional regulation of ACTN2 by PRDM9 was validated through ChIP-qPCR, and the role of the PRDM9-H3K4me3-ACTN2 axis was explored using CRISPR-Cas9 experiments. PDLIM1 was screened as an interaction partner of ACTN2, and its role was verified through functional rescue experiments.

**Results:**

α-actinin-2 (ACTN2) was significantly downregulated in IA tissues. Its knockdown exacerbated vascular wall damage, VSMC apoptosis, and the release of inflammatory factors by inhibiting the Hippo pathway. PRDM9 promoted ACTN2 transcription through H3K4me3 modification, and its low expression led to ACTN2 suppression, driving VSMC proliferation inhibition and promoting apoptosis and inflammation. PDLIM1 interacted with ACTN2, and its overexpression reversed the effects of ACTN2 knockdown, which depended on the Hippo-YAP signaling pathway.

**Conclusion:**

This study reveals that PRDM9 regulates ACTN2 expression through epigenetic modifications and interacts with PDLIM1 to mediate VSMC function and aneurysm progression. The study provides a theoretical basis for clinical intervention.

## Introduction

Intracranial aneurysm (IA) is a highly lethal cerebrovascular disease, the rupture of which can lead to subarachnoid hemorrhage, with a high mortality rate (Pontes et al., 2019; Etminan et al., 2020). Currently, the primary treatment modalities for IA include endovascular interventions (such as coil embolization) and surgical procedures (such as clipping) (Pontes et al., 2019). However, these methods only target the formed aneurysms and cannot fundamentally prevent their occurrence and progression. Studies have shown that the pathogenesis of IA involves the combined effects of multiple factors, including abnormal vascular wall structure, inflammatory responses, and extracellular matrix remodeling (Etminan and Rinkel, 2016; Hallikainen et al., 2021). Therefore, in-depth exploration of the molecular mechanisms of IA and the identification of key regulatory factors are of great significance for the development of novel therapeutic strategies.

Vascular smooth muscle cells (VSMCs) are the main components of the vascular wall, and their phenotypic switching (from contractile to synthetic type) plays a critical role in the progression of IA (Wang et al., 2023). During the development of IA, the imbalance in VSMC proliferation, apoptosis, and inflammatory responses leads to the destruction of the vascular wall structure (Miyata et al., 2020). Specifically, abnormal proliferation of VSMCs promotes vascular wall thickening, while increased apoptosis accelerates vascular wall degeneration (Pan et al., 2021; Hu et al., 2022). Additionally, inflammatory factors (such as TNF-α and IL-1β) secreted by VSMCs further exacerbate the inflammatory response in the vascular wall, ultimately leading to aneurysm formation (Zhang et al., 2022). Therefore, regulating the proliferation, apoptosis and inflammatory of VSMCs is an important strategy for intervening in the progression of IA.

The advancement of high-throughput sequencing technology has provided a robust tool for screening differentially expressed genes associated with IA. The Gene Expression Omnibus (GEO) database, as the largest global platform for gene expression data, has been extensively utilized for the identification and validation of disease biomarkers (Jie et al., 2024). Through GEO data analysis, we identified that ACTN2 (α-actinin-2) is significantly downregulated in IA tissues. ACTN2, a member of the actin-binding protein family, plays a critical role in cytoskeletal remodeling and cell motility (Wang et al., 2024). Previous studies have demonstrated that ACTN2 is involved in the regulation of cardiovascular diseases, such as myocardial hypertrophy and atherosclerosis (Yao et al., 2015; Lindholm et al., 2021). However, its function in IA has not been systematically investigated. Therefore, exploring the role of ACTN2 in IA holds significant scientific value.

Epigenetic regulation has gradually garnered attention in the context of IA, with transcription factor-mediated methylation modifications, such as H3K4me3, being a crucial mechanism of gene expression regulation (Huang et al., 2019; Maimaiti et al., 2023). PRDM9 (PR domain zinc finger protein 9) is an important transcription factor that regulates target gene expression through H3K4me3 modification (Baker et al., 2014). Research has shown that PRDM9 plays a pivotal role in cardiovascular diseases (Chen et al., 2018), but whether it regulates ACTN2 expression in IA via methylation modification remains unclear. Additionally, the role of H3K4me3 modification in other aneurysm-related diseases, such as abdominal aortic aneurysm (Chen et al., 2025), has been preliminarily confirmed, providing a theoretical foundation for exploring the regulatory mechanism of the PRDM9-ACTN2 axis in IA.

Postsynaptic density protein 95, Discs large, Zonula occludens-1 (PDZ) and LIM domain protein 1 (PDLIM1) encodes a member of the enigma protein family. This protein contains two protein interaction domains: a PDZ domain at the N-terminus and one to three LIM domains at the C-terminus (Bauer et al., 2000). It is a cytoskeleton-associated cytoplasmic protein that may function as an adaptor, bringing other LIM-interacting proteins to the cytoskeleton (Bauer et al., 2000). PDLIM1 has been reported to be involved in tumor cell proliferation, migration, invasion, apoptosis, and inflammation, with implicated signaling pathways including the Wnt and Hippo-YAP pathways (Huang et al., 2020). Recently, it was reported that low expression of PDLIM1 promotes the progression of IA.

This study aims to elucidate the role of ACTN2 in IA and its regulatory mechanisms. Through GEO database screening and experimental validation, we found that ACTN2 is significantly downregulated in IA and further demonstrated that it regulates the proliferation, apoptosis, and phenotypic switching of vascular smooth muscle cells (SMCs) by interacting with PDLIM1 (PDZ and LIM domain protein 1). Additionally, we revealed for the first time that PRDM9 transcriptionally regulates ACTN2 through H3K4me3 modification, thereby influencing the progression of IA. This study not only provides new insights into the molecular mechanisms of IA but also lays a theoretical foundation for the development of ACTN2-targeted therapeutic strategies.

## Materials and methods

### Data acquisition and differential gene analysis

The datasets GSE54083 and GSE75436 were downloaded from the GEO database,^1^ GSE54083 was conducted utilizing the GPL4133, manufactured by Agilent Technologies, Inc., located in Santa Clara, CA, USA. The dataset comprised a total of 18 samples, which included 8 samples of ruptured intracranial aneurysms (RIA) and 10 samples of superficial temporal arteries (STA). The RIA samples were exclusively derived from female patients, with ages ranging from 28 to 88 years. In contrast, the STA samples consisted of 8 female and 2 male donors, aged between 34 and 61 years. All tissue specimens were surgically obtained at Tokyo Metropolitan Fuchu Hospital (Tokyo, Japan). Prior to participation in the study, each patient provided written informed consent, which was reviewed and approved by the Ethics Committee of Tokyo Metropolitan Fuchu Hospital, Tokyo Women’s University (Tokyo, Japan). GSE75436 was analyzed using the GPL570 (Affymetrix, Inc., Santa Clara, CA, USA). The dataset comprised 15 IA wall tissues and 15 matched control superficial temporal artery walls. All tissue specimens were surgically obtained at Beijing Tian Tan Hospital, Capital Medical University (Beijing, china), which was approved by the Ethics Committee of Beijing Tian Tan Hospital, Capital Medical University.

The differential gene analysis was performed using the R programming language to identify significantly differentially expressed genes. The specific steps were as follows: raw data were normalized, and batch effects were removed. The limma package was used for differential expression analysis, and significantly differentially expressed genes were screened (|log2FC| > 1, *p* < 0.05). The intersection of differentially expressed genes from multiple datasets was taken to identify genes commonly differentially expressed in IA by Venn analysis. GO analysis was used to classify and annotate the functions of the intersecting genes, describing the functions of genes and gene products from three aspects: molecular function, cellular component, and biological process. KEGG was used to analyze the associations between the information of intersecting genes and biological system networks such as metabolic pathways and disease pathways. These two types of analyses were performed using the clusterProfiler package in R language.

### Human tissue samples

Written informed consent was obtained from all participants prior to the commencement of this study. The study protocol was reviewed and approved by the Ethics Committee of the Affiliated Brain Hospital of Nanjing Medical University (IACUC-204113), in accordance with the ethical principles for medical research involving human subjects as outlined in the Helsinki Declaration. This study included patients diagnosed with intracranial aneurysm who underwent surgical intervention between February 2024 and December 2024. The cohort comprised 20 patients (12 males and 8 females), aged 35–76 years, with a mean age of 53.65 ± 8.77 years (mean ± standard deviation, SD). All cases were confirmed by digital subtraction angiography. Additionally, 20 normal tissue samples were collected from volunteers with traumatic brain injury (11 males and 9 females), aged 33–77 years, with a mean age of 51.56 ± 9.81 years. Excised tissues were promptly frozen in liquid nitrogen and stored at −80°C for subsequent analysis.

### Animal experiments

The Sprague-Dawley rats used in this study were obtained from SiPeiFu (Suzhou) Biotechnology Co., Ltd., [SCXK (SU) 2022-0006]. Upon arrival, the rats were acclimatized to the laboratory environment for 1 week before the commencement of the experiments. They were housed in standard cages under controlled conditions, including a 12-h light/dark cycle, a temperature of 22°C ± 2°C, and a relative humidity of 50% ± 10%. The animals had free access to food and water throughout the study period. After anesthesia, animals were immobilized, and the neck skin was disinfected. Under a stereomicroscope, the neck was gradually incised to expose the trachea, and the common carotid artery was isolated and ligated. Postoperative recovery was monitored, and animals were returned to their cages for continued housing. Two weeks later, animals were re-anesthetized, and the lower abdomen was disinfected. The skin was longitudinally incised to expose the abdominal aorta, and the posterior branches of the left and right renal arteries were ligated. The incision was sutured layer by layer, and postoperative conditions were observed. Animals were continuously monitored and randomly grouped. Sham group (*n* = 10): Only surgical procedures were performed without establishing the aneurysm model. Specific steps included neck incision, trachea exposure and suturing, as well as abdominal incision, abdominal aorta exposure and suturing, but without ligation of the common carotid artery or the posterior branches of the renal arteries. IA group (*n* = 10): The aneurysm model was established without ACTN2 intervention. IA + sh-ACTN2 (*n* = 10): The aneurysm model was established, and ACTN2 knockdown intervention was performed. IA + OE-ACTN2 (*n* = 10): The aneurysm model was established, and ACTN2 overexpression intervention was performed. Prepared lentiviruses with ACTN2 knockdown and overexpression were kept on ice for use. According to the grouping, 50 μL of the virus was injected via the tail vein. If the rats exhibited mild hemiplegia symptoms (such as slow movement, poor symmetry in movement, and weak extension of the unilateral forelimb) along with oculomotor nerve palsy (manifested as deviation of the eye to one side and limited eye rotation), accompanied by increased intracranial pressure and elevated body temperature, the model was considered successfully established. If no such symptoms were observed, animals were continued to be housed. At the experimental endpoint, euthanasia is carried out by using an excessive amount of anesthetics, and Circle of Willis tissues were harvested. Tissues were either frozen or fixed based on subsequent experimental requirements. All experimental procedures involving animals were conducted in strict accordance with the guidelines set forth by the Institutional Animal Care and Use Committee (IACUC). Ethical approval for the study was obtained from the ethics committee of The Affiliated Brain Hospital of Nanjing Medical University under protocol number [IACUC-2406013].

### Hematoxylin and eosin (HE) staining

Tissues were sequentially dehydrated in formalin, 75% ethanol, 85% ethanol, 95% ethanol, and 100% ethanol, followed by clearing in xylene and embedding in paraffin. During embedding, tissue blocks were placed in molten paraffin, cooled, and solidified, and the wax blocks were trimmed. Sections were cut using a microtome, spread at 43°C–45°C, and mounted on slides, which were then baked at 65°C for 30 min. After dewaxing, sections were rehydrated through a graded series of 100% ethanol, 95% ethanol, 85% ethanol, and 75% ethanol. Sections were stained with Harris hematoxylin (Catalog No. G1120, Solarbio, China) for 7 min, rinsed briefly with tap water, differentiated in 1% hydrochloric acid for 5 s, and blued in tap water for 30 min. Subsequently, sections were stained with 0.5% eosin for 30 s, dehydrated through 95% ethanol and 100% ethanol, cleared in xylene, and finally mounted with neutral resin. Staining results were observed under a light microscope (Leica-DM2500, Leica Microsystems GmbH, Wetzlar, Germany).

### Elastica van gieson (EVG) staining

Paraffin sections were subjected to heat treatment for 50 min, followed by dewaxing in xylene for 20 min and rehydration through a graded ethanol series to distilled water, with each step lasting 5 min. Sections were then treated with freshly prepared Weigert’s oxidant for 3 min and rinsed with distilled water for 1 min. Next, sections were bleached with Weigert’s bleach for 3 min and rinsed with distilled water for 1 min. Sections were immersed in Weigert’s resorcin-fuchsin staining solution and stained at room temperature for 1–3 h or at 56°C for 1 h. Differentiation was performed using acidic differentiation solution until no dye was shed, and the reaction was stopped by rinsing with distilled water. Sections were then stained with modified VG staining solution (Catalog No. R20390, Yeasen Biotechnology Co., Ltd., China) for 5–10 min and rinsed with distilled water for 30 s. Finally, sections were dehydrated through 75%, 85%, 95%, and 100% ethanol (3–5 s each), cleared in xylene twice (1 min each), and mounted with neutral resin. Staining results were observed under a light microscope (Leica-DM2500, Leica Microsystems GmbH, Wetzlar, Germany).

### Immunohistochemical staining

Tissues were sequentially treated with formalin for 1 h, 75% ethanol for 100 min, 85% ethanol for 80 min, 95% ethanol for 80 min, 100% ethanol for 1 h (twice), xylene for 40 min, and paraffin for 1 h for dehydration. Molten paraffin was poured into embedding molds, and wax-immersed tissue blocks were placed face-down into the molten paraffin using warmed forceps, gently pressed to ensure contact with the bottom of the mold, and allowed to cool and solidify. Sections were cut using a microtome, spread at 43°C–45°C, and mounted on slides, which were then baked at 65°C for 30 min. After dewaxing, sections were treated with xylene (I) for 15 min, xylene (II) for 10 min, 100% ethanol (I) for 5 min, 100% ethanol (II) for 5 min, and 95% ethanol for 5 min. Antigen retrieval was performed using the citrate antigen retrieval method, and sections were cooled to room temperature and washed with PBS three times (5 min each). Sections were blocked with immunostaining blocking solution at room temperature for 15 min, and the blocking solution was removed. Primary antibody [Anti-ACTN2 (68223-1-Ig, Proteintech), Anti-PDLIM1 (11674-1-AP, Proteintech), or anti-H3K4me3 (Ab8580, Abcam)] was added and incubated at room temperature for 1 h, followed by three PBS washes (5 min each). Secondary antibody [Goat Anti-Rabbit IgG (SA00001-2, Proteintech) or Goat Anti-Mouse IgG (SA00001-1, Proteintech) was incubated for 1 h, followed by three PBS washes (5 min each). DAB (C02-03001, Beyotime) was used for color development, and the endpoint was determined under a microscope. After color development, nuclei were counterstained with hematoxylin for 2 min, blued in tap water, dehydrated through a graded ethanol series, cleared in xylene, and mounted with neutral resin.

### Immunofluorescence staining

Fresh tissue samples were immediately fixed in 4% paraformaldehyde after collection, followed by tissue dehydration through sequential treatments: formalin for 1 h, 75% ethanol for 100 min, 85% ethanol for 80 min, 95% ethanol for 80 min, 100% ethanol for 1 h (twice), xylene for 40 min, and paraffin for 1 h. Molten paraffin was poured into embedding molds, and wax-immersed tissue blocks were placed face-down into the molten paraffin using warmed forceps, gently pressed to ensure contact with the bottom of the mold, and allowed to cool and solidify. Sections were cut using a microtome, spread at 43°C–45°C, and mounted on slides, which were then baked at 65°C for 30 min. After dewaxing, sections were treated with xylene (I) for 15 min, xylene (II) for 10 min, 100% ethanol (I) for 5 min, 100% ethanol (II) for 5 min, and 95% ethanol for 5 min. Antigen retrieval was performed using the citrate antigen retrieval method, and sections were cooled to room temperature and washed with PBS three times (5 min each). Sections were blocked with cold methanol for 10 min, washed with PBS three times (5 min each), permeabilized with 0.5% Triton X-100 for 10 min, and blocked with BSA for 1 h (the serum source must match the secondary antibody animal source). After removing the blocking serum, primary antibody was added and incubated overnight at 4°C, followed by rewarming at room temperature for 30 min and three PBS washes (10 min each). Two diluted fluorescent secondary antibodies (1:200 dilution in antibody dilution buffer) were added and incubated at room temperature in the dark for 1 h, followed by three PBS washes (10 min each). Nuclei were counterstained with DAPI for 10 min, washed with PBS three times (10 min each), and mounted with anti-fade mounting medium. Staining results were observed under a fluorescence microscope.

### TUNEL staining

Sections were dewaxed in xylene for 5–10 min, followed by fresh xylene for another 5–10 min, and then sequentially treated with absolute ethanol for 5 min, 90% ethanol for 2 min, 70% ethanol for 2 min, and distilled water for 5 min. A 20 μg/mL DNase-free proteinase K solution (diluted 1,000-fold in 10 mM Tris-HCl pH 7.4–7.8) was added, and sections were incubated at 20°C–37°C for 15–30 min. Sections were washed with PBS or HBSS three times. Endogenous peroxidase activity was inactivated by incubating sections in 3% hydrogen peroxide in PBS at room temperature for 20 min, followed by three PBS or HBSS washes. Fifty microliters of TUNEL detection solution (A211-01, Vazyme) were added to each sample, and sections were incubated at 37°C in the dark for 60 min, followed by three PBS or HBSS washes. Nuclei were counterstained with DAPI for 10 min, washed with PBS or HBSS three times, and mounted with anti-fade mounting medium. Staining results were observed under a fluorescence microscope.

### Cell culture

Human aortic vascular smooth muscle cells (HAVSMCs) were obtained from Procell Life Science & Technology Co., Ltd., (CP-H116, Wuhan, Hubei, China). The cells were cultured in Dulbecco’s Modified Eagle’s Medium (DMEM) (Catalog No. 11965092, Thermo Fisher Scientific, Carlsbad, CA, USA), supplemented with 10% fetal bovine serum (FBS) (Catalog No. 10099141C, Thermo Fisher Scientific, Waltham, MA, USA) and 1% penicillin/streptomycin (Catalog No. 15140122, Thermo Fisher Scientific, Carlsbad, CA, USA). The cells were maintained at 37°C in a humidified atmosphere containing 5% CO_2_.

### CCK8 assay

First, 100 μL of 2,000 cells were added to each well, and cells were seeded into a 96-well plate (100 μL/well, avoiding bubble formation) according to experimental requirements, with a blank control group set up. After pre-culturing for 24 h, cells were transfected according to group requirements. Ten microliters of CCK-8 Solution (C0037, Beyotime) were added to each well, and plates were incubated in a cell culture incubator for 2–4 h. Finally, the absorbance at 450 nm was measured using a microplate reader, and cell viability was calculated.

### qRT-PCR

First, cells were collected, and total RNA was extracted using an RNA extraction kit. The concentration and purity of RNA were measured using a micro nucleic acid and protein analyzer. Subsequently, RT-PCR was performed: according to the instructions of the Vazyme reverse transcription kit, 4 μL of 5 × ATGScript^®^ RT Mix, 2 μL of ATGScript^®^ Enzyme Mix, 1 μL of Oligo (dT), 1 μg of total RNA, and 12 μL of RNase-free ddH2O were added to a 1.5 mL centrifuge tube. The reaction was carried out at 55°C for 15 min, followed by 85°C for 5 s to complete reverse transcription. The quantitative PCR amplification system included 8.2 μL of H_2_O, 10 μL of 2 × ATGStart^®^ qPCR SYBR Green Master Mix, 0.4 μL of Primer1, 0.4 μL of Primer2, and 1 μL of cDNA. The reaction conditions were pre-denaturation at 95°C for 8 min, followed by 40 cycles of PCR (95°C for 10 s, 60°C for 30 s, and 56°C for 5 s), with a melting curve collected from 60°C to 95°C. Finally, Ct values were obtained using real-time quantitative PCR, and the relative gene expression levels were calculated using the 2^–△△Ct^ method. The primer sequences used included ACTN2, PRDM9, PDLIM1, SM22α, αSMA, and GAPDH.

ACTN2-F: GTCCCTGACGGAAGTTCGAG

ACTN2-R: CCTGAGCAATGGCTGCAATC

PRDM9-F: CCAGGAGCATCTGCAAGGAT

PRDM9-R: GACTTCATTGCTGGCTTTGTCA

PDLIM1-F: CCCCAAGAAGTCCTGCACAT

PDLIM1-R: GAGCAGGTGAGGCGGTAAAT

GAPDH-F: GAAGGTGAAGGTCGGAGTC

GAPDH-R: GAAGATGGTGATGGGATTTC

### Western blot

The rat Circle of Willis were collected and homogenized in lysis buffer. Protein standard and BCA working solutions were prepared according to the BCA kit instructions, added to a 96-well plate, and incubated at 37°C for 20–30 min. The absorbance at 562 nm was measured using a microplate reader to calculate protein concentration. Protein samples were mixed with SDS-PAGE loading buffer, boiled for denaturation, and stored. A 10% separating gel and a stacking gel were prepared and allowed to solidify at room temperature. A 10X electrophoresis buffer and transfer buffer were prepared and diluted for use. Protein samples were loaded into the gel wells, and electrophoresis was performed at 85 V until the samples entered the separating gel, then adjusted to 130 V until the samples approached the bottom. PVDF membranes were activated with methanol, and the transfer sandwich was assembled. Transfer was performed at a constant current of 310 mA for 60 min. After transfer, membranes were blocked with 5% skim milk for 1 h, incubated with primary antibody [Anti-ACTN2 (68223-1-Ig, Proteintech), Anti-PDLIM1 (11674-1-AP, Proteintech), Anti-YAP1 (81090-1-RR, Proteintech), Anti-CTGF (23903-1-AP, Proteintech), Anti-CYR61 (26689-1-AP, Proteintech), Anti-Caspase-3 (82202-1-RR, Proteintech), Anti-Cleaved caspase-3 (25128-1-AP, Proteintech), Anti-PARP (ab191217, Abcam), Anti-Cleaved PARP (ab32064, Abcam), Anti-PRDM9 (ab191524, Abcam), Anti-GAPDH (ab9485, Abcam)] at 4°C overnight, washed with TBST, and incubated with secondary antibody goat anti-rabbit IgG antibody (ab6721, Abcam) or rabbit anti-mouse IgG antibody (ab6728, Abcam) at room temperature for 60 min. After washing, ECL chemiluminescence was used for detection.

### RNA immunoprecipitation (RIP)

Human aortic vascular smooth muscle cells were washed twice with pre-chilled PBS and dried. Pre-chilled RIPA Buffer was added, and cells were scraped off and transferred to a 1.5 mL EP tube. The suspension was gently shaken at 4°C for 15 min and centrifuged at 14,000 *g* at 4°C for 15 min. The supernatant was transferred to a new EP tube. Protein A + G agarose beads were washed twice with PBS and prepared to a 50% concentration. For every 1 mL of total protein, 100 μL of agarose beads and 200 U/mL of RNase Inhibitor were added, and the mixture was shaken at 4°C for 10 min. The mixture was centrifuged at 14,000 *g* at 4°C for 15 min, and the supernatant was transferred to a new EP tube. Total protein was diluted to approximately 1 μg/μL with PBS, and rabbit anti-antibody was added. The mixture was gently shaken at 4°C overnight. Then, 100 μL of Protein A + G agarose beads and 200 U/mL of RNase Inhibitor were added, and the mixture was gently shaken at 4°C overnight or at room temperature for 1 h. The mixture was centrifuged at 14,000 rpm for 5 s, and the agarose bead-antibody-antigen complex was collected. The complex was washed three times with pre-chilled RIPA Buffer or PBS (800 μL each time). Finally, RNA bound to the beads was extracted.

### Cas9-sgRNA knockout

In accordance with the manufacturer’s protocol, we utilized the Dharmacon Edit-R CRISPR-Cas9 Gene Engineering System with Lentiviral Cas9 Nuclease (Dharmacon Edit-R™) for our experiments. The specific procedures are as follows: Initially, VSMCs cells were infected with the lentiviral Cas9 nuclease. Subsequently, cells expressing Cas9 were selected using blasticidin, and single clones were isolated and expanded. Next, inducible Cas9 clones were prepared using lentiviral ACTN2-sgRNA (Dharmacon Edit-R™, source clone ID VSGH12608-256887302). Cells were then screened with puromycin, and the knockout of the ACTN2 gene was induced by adding doxycycline.

### RNA pull-down

The Pierce™ RNA 3′ End Desthiobiotinylation Kit was used to label RNA with biotin probes. RNA was mixed with DMSO, denatured at 85°C for 5 min, and immediately placed on ice for 5 min. The probe labeling reaction system was prepared and incubated at 16°C for 4 h to overnight. After the reaction, RNA was extracted using phenol-chloroform and precipitated with ethanol overnight. After centrifugation, the precipitate was washed with 70% ethanol, dried, and dissolved in nuclease-free water. The RNA was denatured at 95°C for 5 min and stored for use. Cells were washed with pre-chilled PBS and lysed with cell lysis buffer A, followed by three freeze-thaw cycles in liquid nitrogen. The supernatant was collected after centrifugation. Magnetic beads were pre-treated, washed with binding and washing buffer, and combined with RNA, followed by slow rotation at room temperature for 20 min. The beads were washed with cell lysis buffer A, and cell lysate and RNase Inhibitor were added. The mixture was slowly rotated at 4°C for 2 h. The supernatant was removed using a magnetic stand, and the beads were washed five times with cell lysis buffer A. Finally, RNA bound to the beads was extracted and verified using qPCR.

### Chromatin immunoprecipitation (ChIP)

Cells in culture dishes were cross-linked with 1% formaldehyde for 10 min, and the reaction was terminated by adding glycine. Cells were washed with PBS containing 1 mM PMSF, scraped off, aliquoted, and centrifuged to form a pellet. The pellet was resuspended in SDS Lysis Buffer containing 1 mM PMSF and lysed on ice for 10 min, followed by sonication to fragment genomic DNA into 100–1000 bp pieces. After sonication, the sample was centrifuged, and the supernatant was collected and diluted with ChIP Dilution Buffer. A 20 μL aliquot was taken as the Input control, while the remaining sample was pre-cleared with Protein A + G Agarose/Salmon Sperm DNA for 30 min. After centrifugation, the supernatant was incubated with primary antibody at 4°C overnight. Protein A + G Agarose/Salmon Sperm DNA was then added to capture the antibody-protein-DNA complex. The complex was sequentially washed with Low Salt, High Salt, LiCl, and TE Buffer. The complex was eluted with Elution Buffer, and the eluates were combined. NaCl was added, and the mixture was heated at 65°C for 4 h to reverse cross-linking. EDTA, Tris, and proteinase K were added, and the mixture was incubated at 45°C for 60 min. DNA was extracted using phenol-chloroform, precipitated with ethanol, washed, and resuspended in TE or water for PCR detection of target genes.

### EdU assay

Cells were cultured in a 12-well plate and allowed to recover overnight. After the required treatment, a 2X EdU working solution (C0071S, Beyotime) was prepared and added to the wells in equal volume. Cells were incubated for 10% of the cell cycle time in the incubator. After incubation, the culture medium was removed, and cells were fixed with 10% paraformaldehyde at room temperature for 15 min, followed by three PBS washes. Cells were permeabilized with PBS containing 0.3% Triton X-100 at room temperature for 10–15 min and washed once or twice with washing buffer. The Click Additive Solution reaction mixture was prepared, added to the wells, and incubated at room temperature in the dark for 30 min, followed by three PBS washes. A 1X Hoechst 33342 solution was prepared, added to the wells, and incubated at room temperature in the dark for 10 min, followed by three washes with washing buffer. Finally, fluorescence detection was performed to assess cell proliferation.

### Flow cytometry

Cells were collected and resuspended in cell washing buffer to a concentration of 1 × 107/mL, followed by centrifugation at 500 *g* and 4°C for 5 min. Cells were washed twice with pre-chilled 1 × PBS and centrifuged at 500 *g* and 4°C for 5 min. The cell pellet was resuspended in 100 μL of pre-chilled Buffer, followed by the addition of 5 μL Annexin V-FITC and 5 μL PI. The mixture was gently mixed and incubated at room temperature in the dark for 15 min. Then, 400 μL of pre-chilled Buffer was added, and the mixture was gently mixed. The sample was kept on ice in the dark. The flow cytometer was turned on, flushed, and the appropriate channels were selected. The sample was then analyzed to assess cell apoptosis.

### ELISA

Antigen or antibody was diluted in coating buffer and added to the wells of an ELISA plate, which was then coated overnight at 4°C. The coating solution was discarded, and the plate was washed three times with washing buffer for 3–5 min each. Blocking buffer was added to block non-specific binding sites, and the plate was incubated at 37°C for 1–2 h, followed by three washes with washing buffer. Test samples or standards were added to the wells, and the plate was incubated at 37°C for 1–2 h, followed by three washes with washing buffer. Enzyme-labeled antibody was added, and the plate was incubated at 37°C for 1 h, followed by three washes with washing buffer. Substrate solution was added, and the plate was incubated at room temperature in the dark for 10–30 min for color development. Stop solution was added to terminate the reaction, and the absorbance of each well was immediately measured using a microplate reader. The concentration of the target protein in the test samples was calculated based on the standard curve.

### Statistical analyses

The statistical analysis was conducted using SPSS 22.0 software. Data are presented as mean ± standard deviation (SD). For comparisons between two groups, significant differences were determined using factorial analysis with Student’s *t*-test. For comparisons involving three or more groups, one-way ANOVA followed by Tukey’s *post-hoc* test was applied. To control for potential Type I errors due to multiple comparisons, we implemented the Benjamini-Hochberg procedure for false discovery rate (FDR) correction. A *P*-value of less than 0.05 was considered statistically significant. For *in vitro* experiments, each experiment was performed in triplicate (three replicates), and the entire experiment was independently repeated three times.

## Results

### Low expression of ACTN2 in IA

**FIGURE 1:**
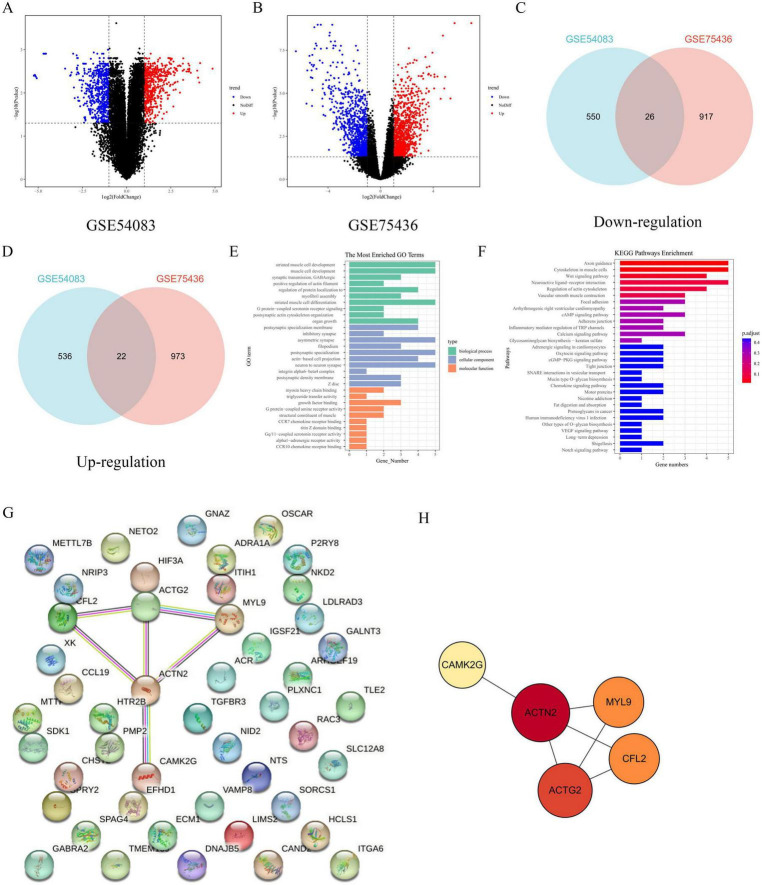
Gene Expression Analysis and Functional Enrichment of IA and STA Tissues. **(A,B)** Volcano plots illustrate gene expression changes in the GSE54083 and GSE75436 datasets. The *x*-axis represents log2 fold change, and the *y*-axis represents the negative log10 *P*-value. Different colors indicate distinct trends: blue for downregulated genes, red for upregulated genes, and black for genes with no significant changes. **(C,D)** Venn diagrams display the number of commonly downregulated and upregulated genes in the two datasets. Panel C shows 550 downregulated genes in GSE54083 and 917 in GSE75436, with 26 genes overlapping. Panel D shows 536 upregulated genes in GSE54083 and 973 in GSE75436, with 22 genes overlapping. **(E)** Bar plot lists the most enriched GO terms among upregulated genes, covering biological processes, cellular components, and molecular functions. **(F)** Bar plot presents KEGG pathway enrichment analysis results, with colors ranging from blue to red indicating increasing enrichment significance (low to high *p*-values). **(G,H)** Network and simplified network diagrams depict gene interactions relevant to the study. Panel G, derived from STRING, illustrates the complex gene interaction network, while Panel H, from Cytoscape, focuses on the key gene ACTN2 and its direct interactions.

**FIGURE 2:**
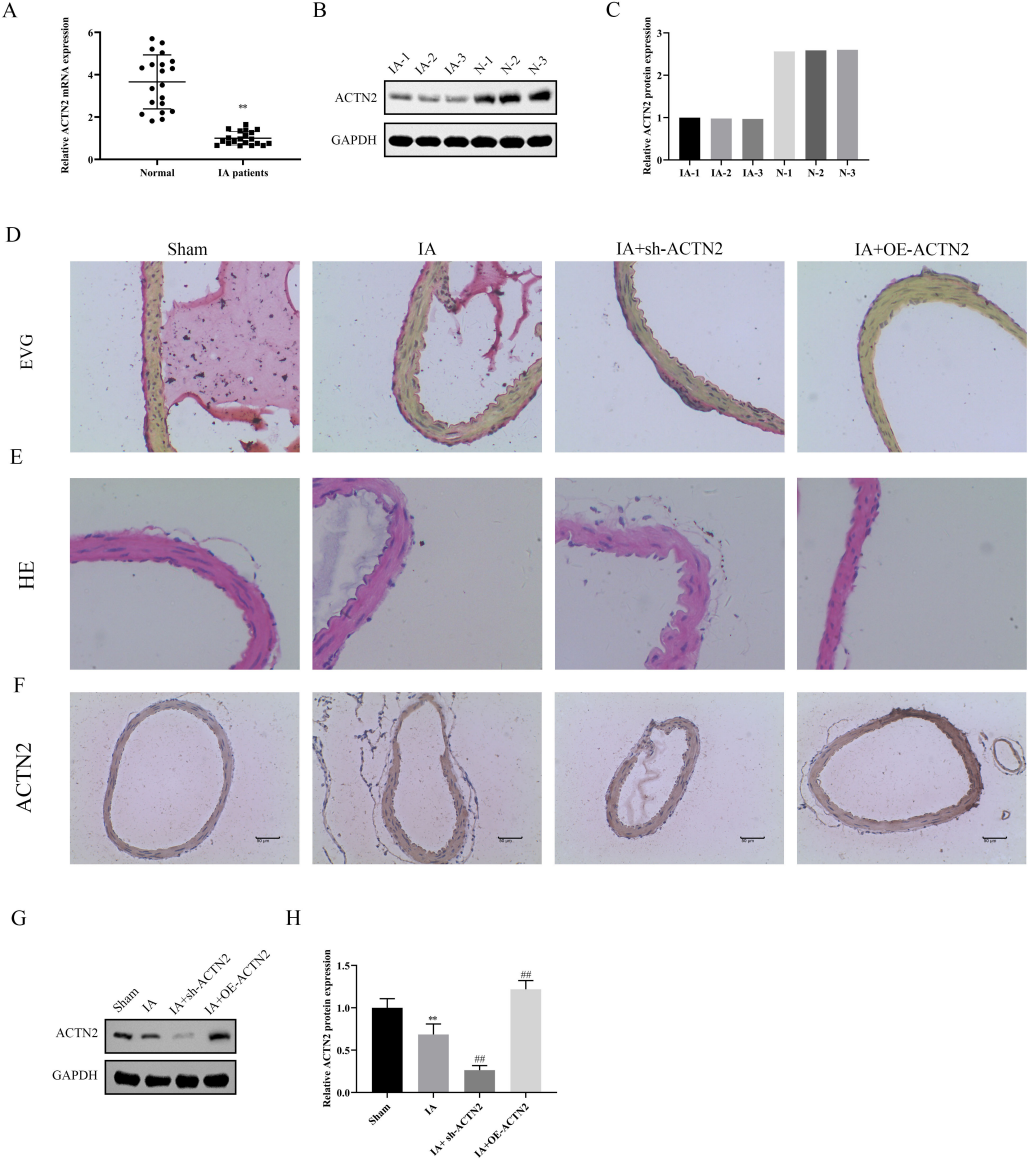
Expression of ACTN2 in IA patients and its impact on vascular structure. **(A)** Relative mRNA expression levels of ACTN2 in STA and IA groups, showing significantly reduced ACTN2 expression in IA patients. **(B,C)** Western blot analysis of ACTN2 protein expression in IA patients and STA groups. **(D–F)** Vascular histological changes in Sham, IA, IA + sh-ACTN, and IA + OE-ACTN groups, visualized by EVG staining **(D)**, HE staining **(E)**, and ACTN2 immunohistochemical staining **(F)**. **(G,H)** Western blot and bar plot analysis of ACTN2 protein expression levels in Sham, IA, IA + sh-ACTN, and IA + OE-ACTN groups. **P < 0.01 vs. Normal, **P < 0.01 vs. Sham, ##*P* < 0.01 vs. IA.

We analyzed the gene expression profiles from two datasets, GSE54083 and GSE75436, to identify differentially expressed genes (DEGs). Volcano plots revealed significant up- and down-regulated genes in both datasets (Figures 1A, B), with 22 common up-regulated genes and 26 common down-regulated genes identified through Venn diagram analysis (Figures 1C, D). Functional enrichment analysis using Gene Ontology (GO) terms showed that the most enriched biological processes, cellular components, and molecular functions among genes included striated muscle cell development, synaptic transmission, GABAergic, post-synaptic specialization membrane, inhibitory synapse, asymmetric synapse, myosin heavy chain binding, triglyceride transfer activity, and G protein-coupled amine receptor activity, indicating potential roles in neuronal function (Figure 1E). KEGG pathway analysis further highlighted pathways such as axon guidance, Wnt signaling pathway, and neurotrophic ligand-receptor interaction, suggesting involvement in cellular communication and development (Figure 1F). A protein-protein interaction (PPI) network was constructed to explore the interactions among DEGs, revealing a central role for ACTN2, which interacted with multiple proteins including MYL9, CFL2, and ACTG2 (Figures 1G, H). These findings suggest that ACTN2 and its interacting partners may play crucial roles in the observed biological processes and pathways, providing insights into the molecular mechanisms underlying the studied condition. qRT-PCR and western blot analyses revealed significantly lower ACTN2 expression in IA patients compared to Normal (Figures 2A–C).

### ACTN2 can alleviate the progression of IA in a mouse model

**FIGURE 3:**
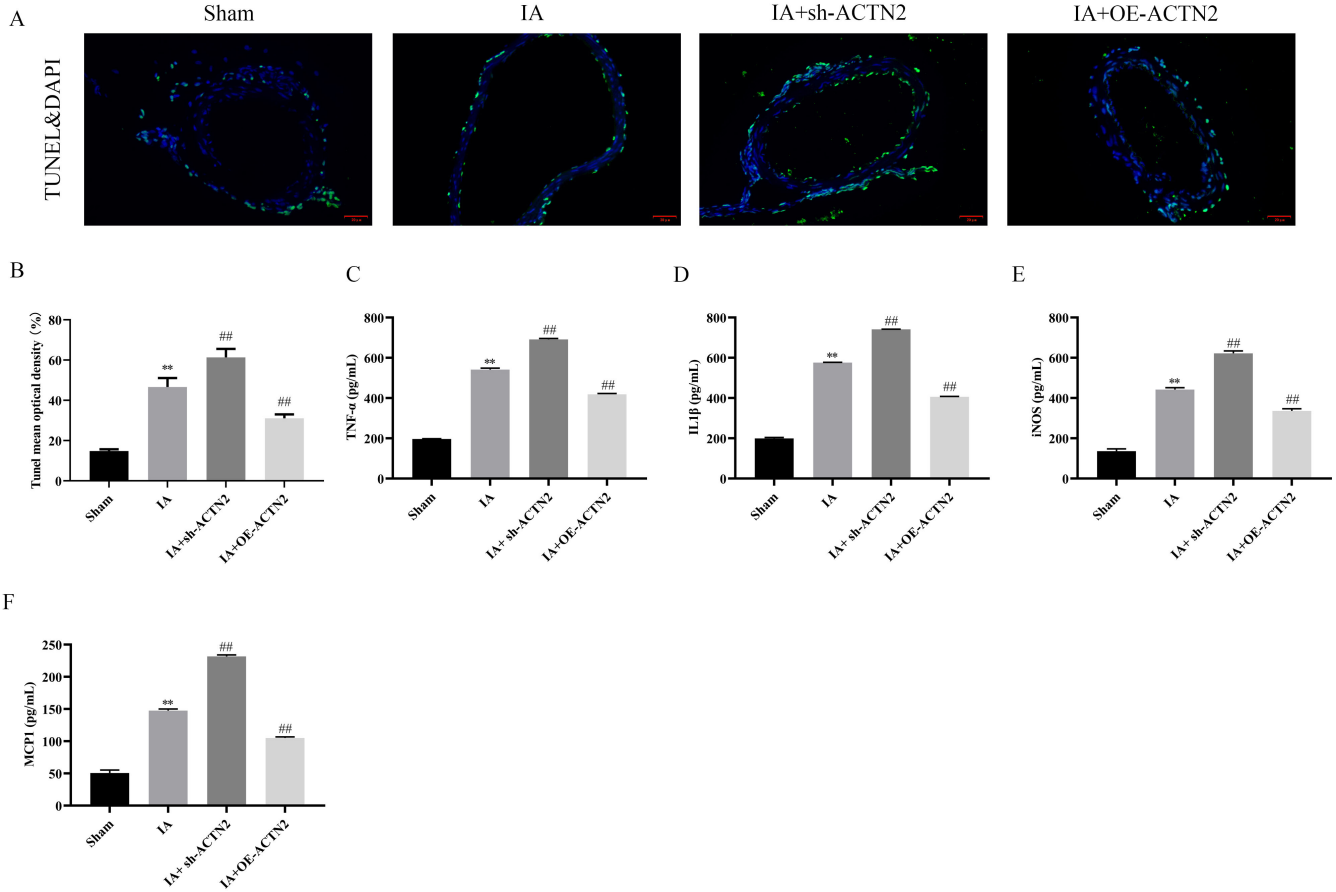
Impact of ACTN2 on Apoptosis and Inflammatory Factor Expression in the IA Model. **(A,B)** TUNEL staining with DAPI nuclear staining shows vascular wall cell apoptosis in Sham, IA, IA + sh-ACTN2, and IA + OE-ACTN2 groups. **(C–F)** Bar plots display the expression levels of inflammatory factors TNF-α, IL-1β, iNOS, and MCP-1 in each group. **P < 0.01 vs. Sham, ##*P* < 0.01 vs. IA.

To further explore the functional implications, we induced cerebral aneurysms in mice using unilateral carotid artery ligation and renal vascular hypertension. Histological analysis via Elastica van Gieson and HE staining demonstrated severe vascular wall lesions, including collagen layer loss, fibrosis, and VSMC thinning in the IA and sh-ACTN2 groups, whereas OE-ACTN2 resulted in milder lesions (Figures 2D, E). Immunohistochemistry and WB confirmed reduced ACTN2 expression in the IA and sh-ACTN2 groups compared to Sham group, with OE-ACTN2 restoring expression (Figures 2F–H). TUNEL assays indicated increased apoptosis in the IA and sh-ACTN2 groups compared to Sham group, while OE-ACTN2 reduced apoptosis (Figures 3A, B). ELISA showed elevated levels of inflammatory cytokines (TNF-α, IL1β, iNOS, MCP1) in the IA and sh-ACTN2 groups, which were attenuated by OE-ACTN2 (Figures 3C–F).

### ACTN2 promotes the proliferation of VSMCs and inhibits cell apoptosis and inflammation

**FIGURE 4:**
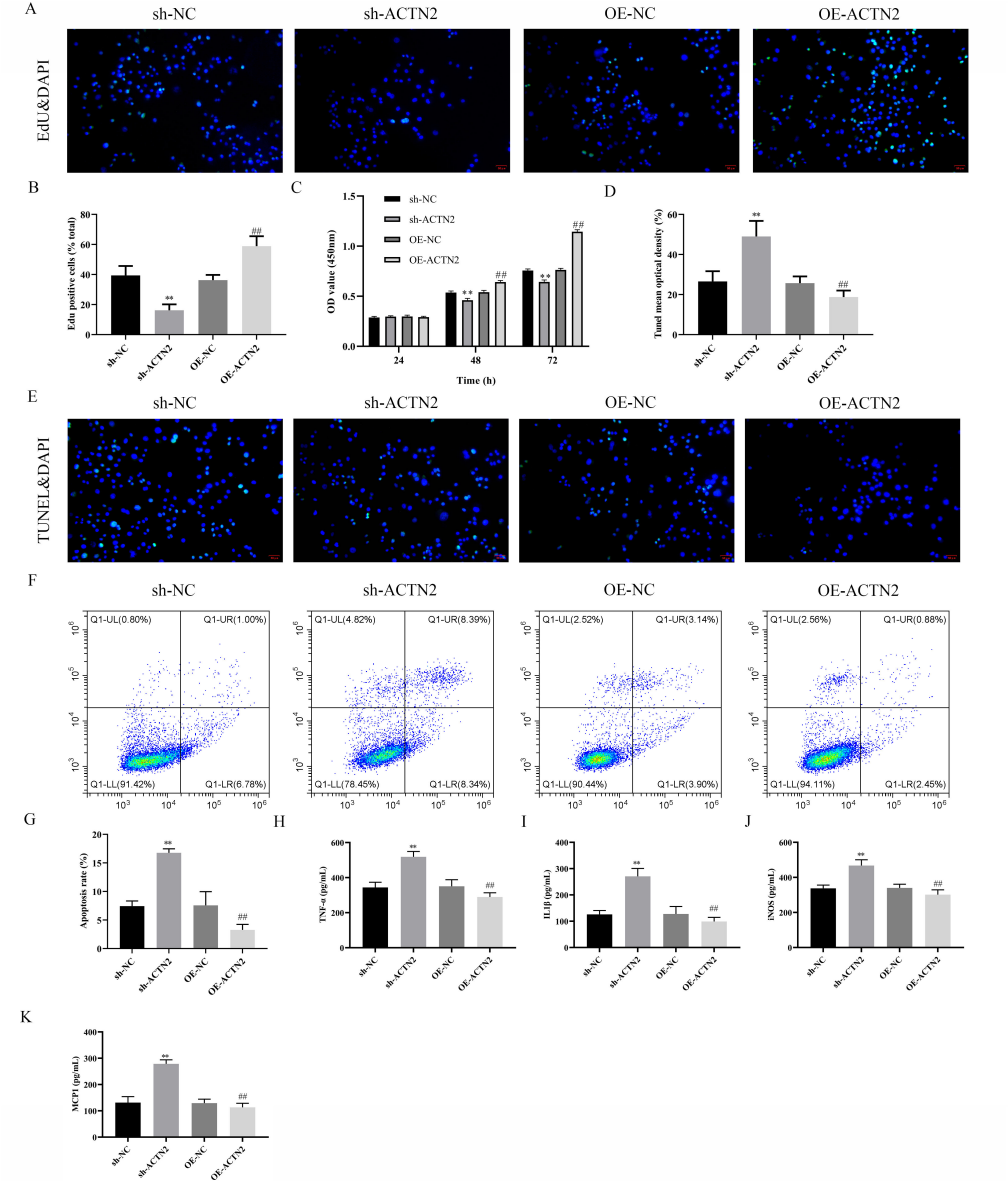
Effects of ACTN2 on Cell Proliferation, Apoptosis, and Inflammatory Factor Expression. **(A,B)** EdU staining with DAPI nuclear staining shows cell proliferation in sh-NC, sh-ACTN2, OE-NC, and OE-ACTN2 groups. **(C)** CCK8 assay measures cell viability at 24 h, 48 h, and 72 h in each group. **(D,E)** TUNEL staining with DAPI nuclear staining illustrates cell apoptosis in each group. **(F,G)** Flow cytometry quantifies the apoptotic proportion of cells in each group. **(H–K)** Bar plots present the expression levels of inflammatory factors TNF-α, IL-1β, iNOS, and MCP-1 in each group. **P < 0.01 vs. sh-NC, ##*P* < 0.01 vs. oe-NC.

**FIGURE 5:**
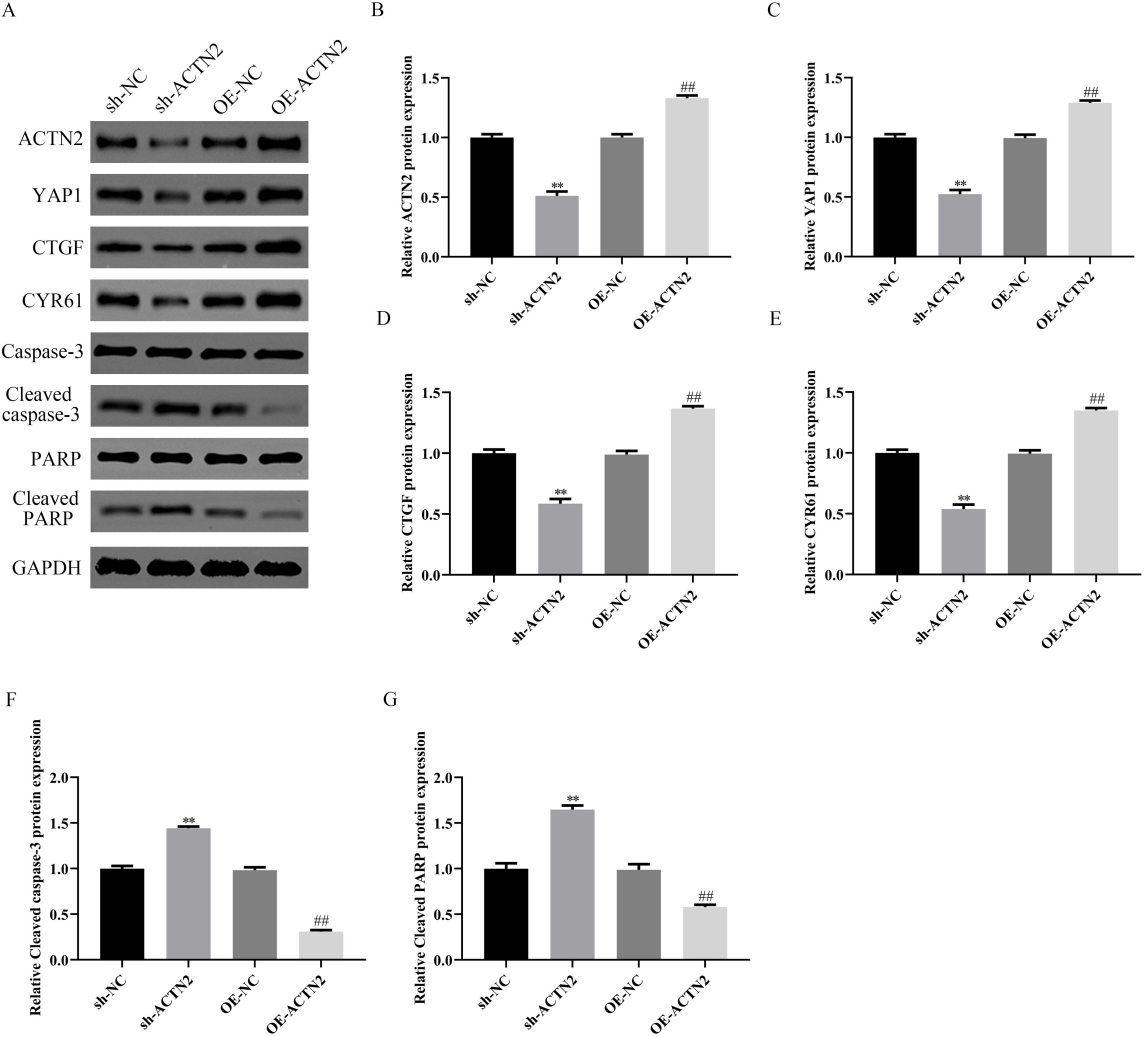
Impact of ACTN2 on Related Protein Expression. **(A)** Western blot analysis of ACTN2, YAP1, CTGF, CYR61, Caspase-3, Cleaved caspase-3, PARP, and Cleaved PARP protein expression in sh-NC, sh-ACTN2, OE-NC, and OE-ACTN2 groups, with GAPDH as the internal control. **(B–G)** Bar plots display the relative expression levels of the aforementioned proteins in each group. **P < 0.01 vs. sh-NC, ##*P* < 0.01 vs. oe-NC.

*In vitro*, CCK-8 and EdU assays demonstrated reduced VSMC proliferation in the sh-ACTN2 group compared to sh-NC group, with OE-ACTN2 enhancing proliferation (Figures 4A–C). Flow cytometry and TUNEL assays confirmed increased apoptosis in the sh-ACTN2 group versus sh-NC group, while OE-ACTN2 reduced apoptosis (Figures 4D–G). ELISA showed increased levels of inflammatory cytokines (TNF-α, IL1β, iNOS, MCP1) in the sh-ACTN2 group and decreased levels in OE-ACTN2 group (Figures 4H–K). WB analysis indicated that ACTN2 knockdown suppressed the Hippo signaling pathway (YAP1, CTGF, CYR61) and increased apoptosis-related proteins (cleaved caspase-3, cleaved PARP), whereas ACTN2 overexpression exerted opposite effects (Figures 5A–G). These findings collectively suggest that low expression of ACTN2 plays a critical role in inhibiting VSMC growth and promoting inflammation in aneurysms.

### PRDM9 transcriptionally regulates ACTN2 and participates in the regulation of the aneurysm process

**FIGURE 6:**
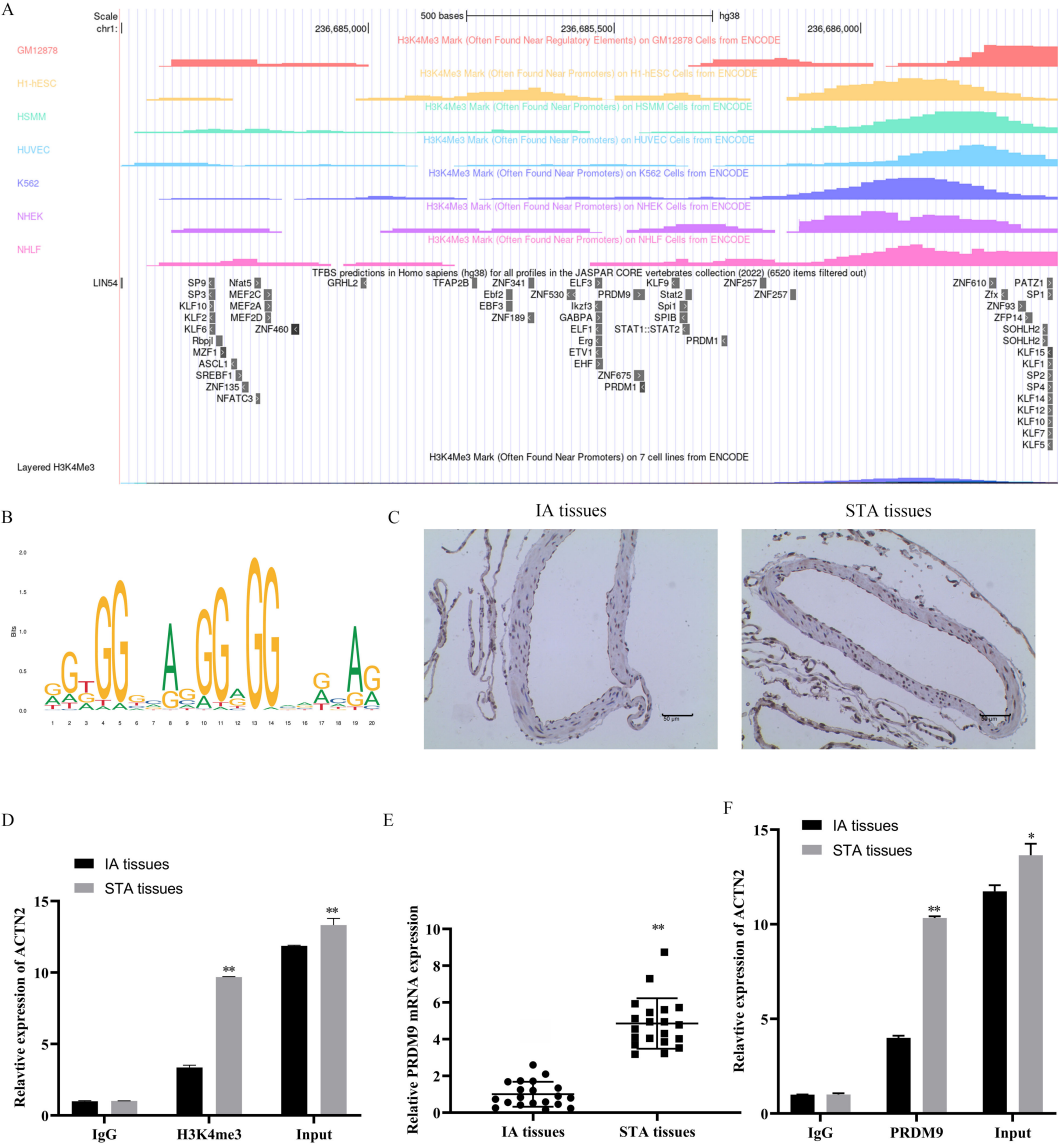
PRDM9-Mediated H3K4me3 Regulation of ACTN2 Gene Expression in IA and STA Tissues. **(A)** H3K4me3 modifications are primarily concentrated near the ACTN2 gene promoter region across different cell lines (GM12878, H1-hESC, HSMM, HUVEC, K562, NHEK, NHLF). Transcription regulatory sites near the ACTN2 promoter, including PRDM9, are also shown. **(B)** The transcription factor binding motif within the ACTN2 promoter was predicted using JASPAR. **(C)** Immunohistochemical staining of IA and STA tissues shows differential expression of H3K4me3. **(D)** ChIP-qPCR analysis confirms the relationship between H3K4me3 and the ACTN2 promoter. **(E)** PRDM9 protein expression is significantly lower in IA tissues compared to STA tissues. **(F)** ChIP-qPCR analysis demonstrates the relationship between PRDM9 and the ACTN2 promoter. *P < 0.05 vs. IA tissues, **P < 0.01 vs. IA tissues.

**FIGURE 7:**
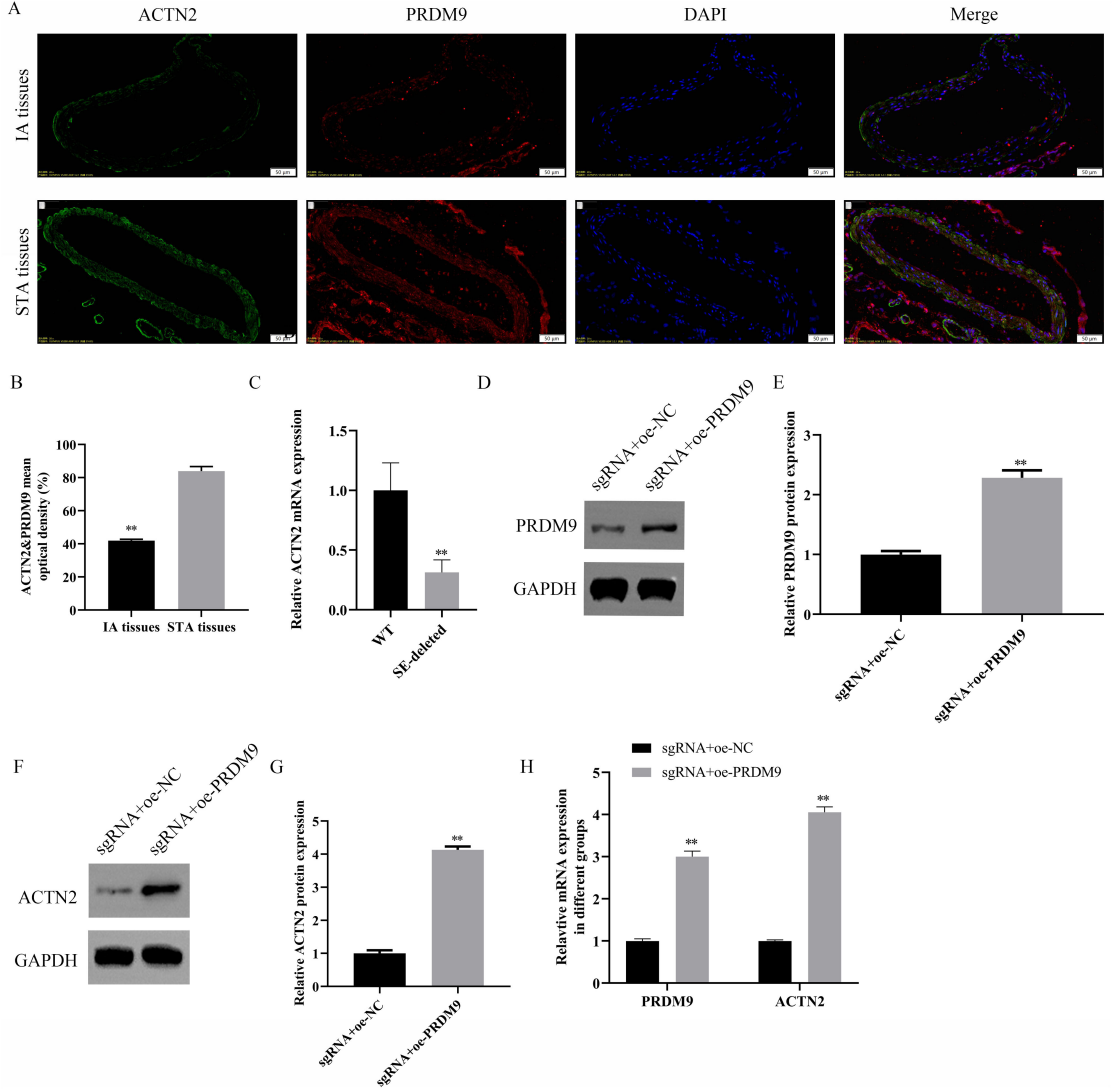
Regulatory Role of PRDM9 on ACTN2 Expression. **(A,B)** Immunofluorescence staining shows the localization of ACTN2 and PRDM9 proteins in IA and STA tissues. ACTN2 is primarily expressed in the cell membrane and cytoplasm of IA tissues, while PRDM9 is mainly localized in the nucleus. DAPI staining marks the nuclei, and merged images illustrate the co-localization of the two proteins in different tissues. **(C)** Bar plot displays the relative mRNA expression levels of ACTN2 in WT and SE-deleted groups. **(D–G)** Western blot analysis of PRDM9 and ACTN2 protein expression in sgRNA + oe-NC and sgRNA + oe-PRDM9 groups. **(H)** Bar plot presents the relative mRNA expression levels of PRDM9 and ACTN2 in sgRNA + oe-NC and sgRNA + oe-PRDM9 groups. **P < 0.01 vs. WT, **P < 0.01 vs. sgRNA + oe-NC.

**FIGURE 8:**
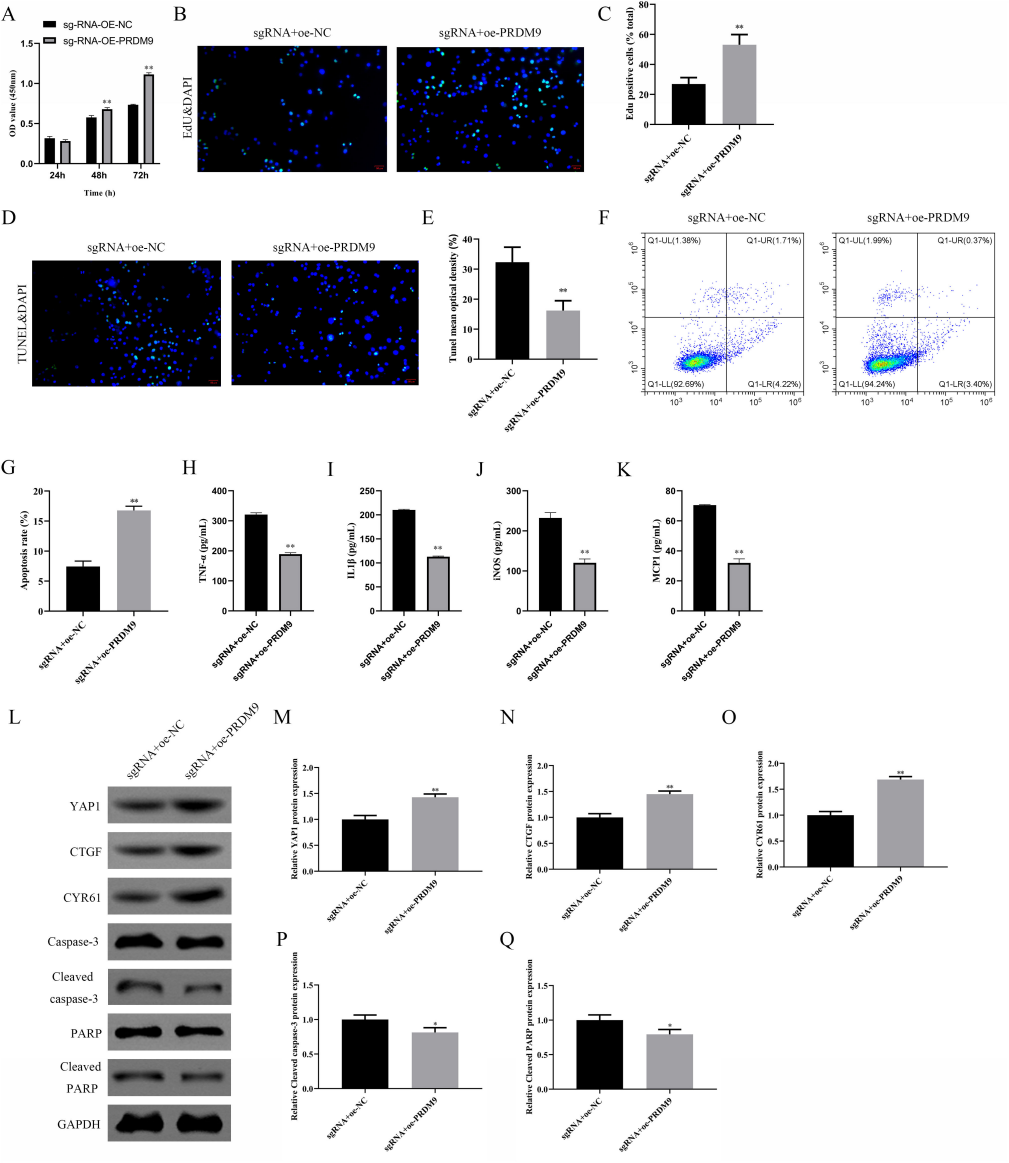
Effects of PRDM9 Overexpression on Cell Proliferation, Apoptosis, Inflammatory Factors, and Related Protein Expression. **(A)** Bar plot shows the OD values at different time points (24 h, 48 h, 72 h) in sgRNA + oe-NC and sgRNA + oe-PRDM9 groups. **(B,C)** EdU staining with DAPI nuclear staining illustrates cell proliferation in the two groups. **(D,E)** TUNEL staining with DAPI nuclear staining demonstrates cell apoptosis in the two groups. **(F,G)** Flow cytometry quantifies the apoptotic proportions, showing significantly lower early and late apoptosis rates in the sgRNA + oe-PRDM9 group compared to the sgRNA + oe-NC group. **(H–K)** Bar plots display the expression levels of inflammatory factors TNF-α, IL-1β, iNOS, and MCP-1 in the two groups. **(L–Q)** Western blot analysis of YAP1, CTGF, CYR61, Caspase-3, Cleaved caspase-3, PARP, and Cleaved PARP protein expression in the two groups. *P < 0.05 vs. sgRNA + oe-NC, **P < 0.01 vs. sgRNA + oe-NC.

We explored the regulatory mechanisms of ACTN2 in aneurysms through epigenetic and transcriptional analyses. Using the USUC database, we predicted H3K4me3 modifications near the ACTN2 promoter (Figures 6A, B), which were validated by immunohistochemistry showing lower H3K4me3 levels in IA tissues compared to STA tissues (Figure 6C). ChIP-qPCR confirmed stronger H3K4me3 binding to the ACTN2 promoter in STA tissues than in IA tissues (Figure 6D). Furthermore, we identified PRDM9 as a transcription factor binding to the ACTN2 promoter via the USUC database (Figure 6A). RT-PCR revealed reduced PRDM9 expression in IA tissues compared to STA tissues (Figure 6E). ChIP-qPCR demonstrated weakened PRDM9 binding to the ACTN2 promoter in IA tissues versus STA tissues (Figure 6F). FISH confirmed the co-localization of ACTN2 and PRDM9, supporting their interaction (Figures 7A, B). We hypothesized that low-expression PRDM9 suppressed H3K4me3, leading to reduced ACTN2 expression and contributing to aneurysm progression. To test this, we used CRISPR-Cas9 to delete the ACTN2 enhancer, resulting in significantly lower ACTN2 expression compared to wild-type (WT) (Figure 7C). Overexpression of PRDM9 in ACTN2-sgRNA cells increased PRDM9 levels and restored ACTN2 expression (Figures 7D–H). Functional assays showed that PRDM9 overexpression enhanced VSMCs proliferation and reduced apoptosis compared to controls (Figures 8A–G). ELISA revealed decreased levels of inflammatory cytokines (TNF-α, IL1β, iNOS, MCP1) in PRDM9-overexpressing cells (Figures 8H–K). WB analysis indicated that PRDM9 overexpression activated the Hippo signaling pathway (YAP1, CTGF, CYR61) and reduced apoptosis-related proteins (cleaved caspase-3, cleaved PARP) (Figures 8L–Q). These findings suggested that PRDM9-mediated suppression of H3K4me3 reduces ACTN2 expression, promoting VSMC dysfunction and aneurysm progression, highlighting potential therapeutic targets for aneurysm treatment.

### Overexpression of PDLIM1 reverses the effects of ACTN2 knockdown on vascular smooth muscle cells

**FIGURE 9:**
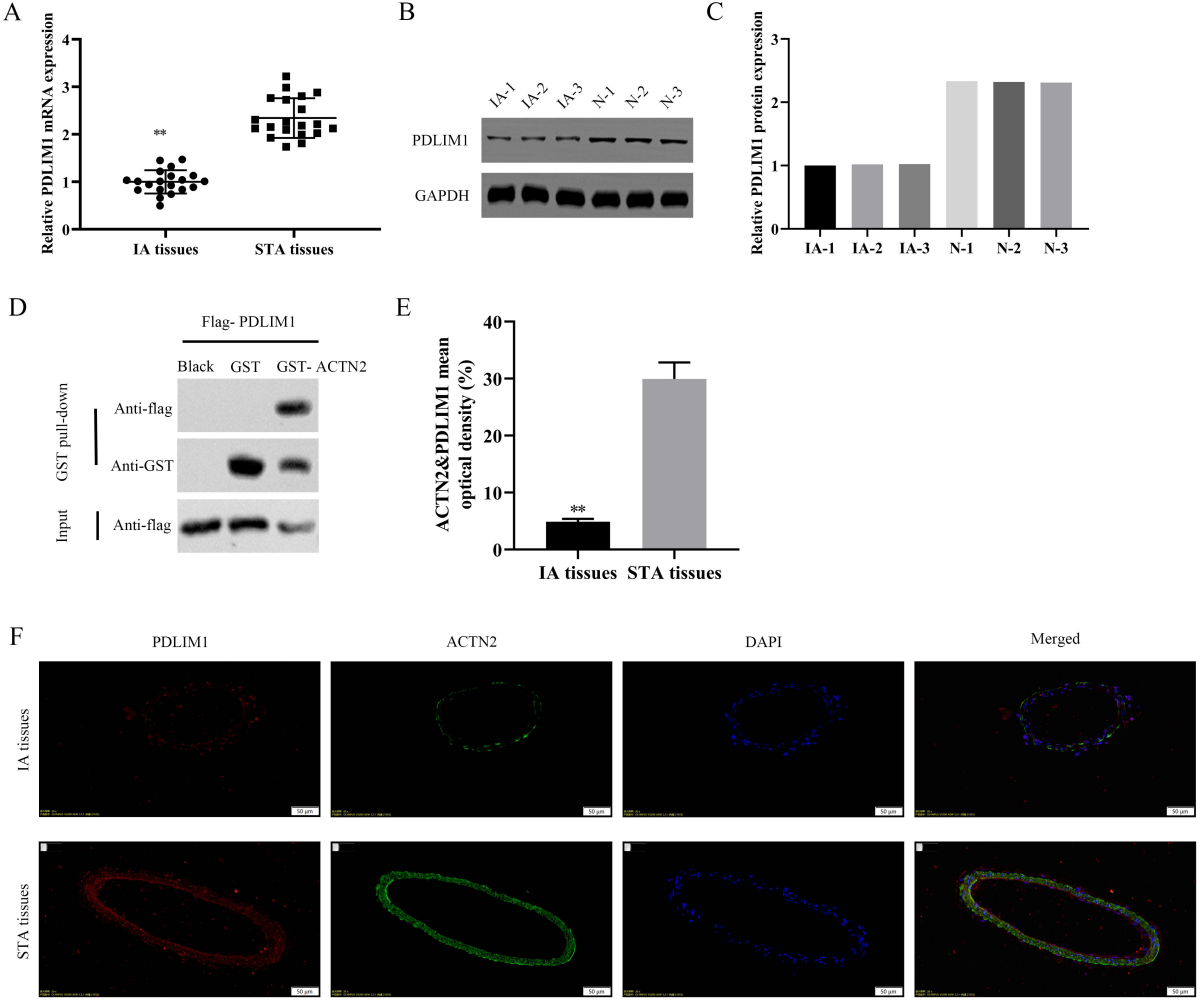
Expression and Interaction of PDLIM1 and ACTN2 in IA and STA Tissues. **(A)** Bar plot shows the relative mRNA expression levels of PDLIM1 in IA and STA tissues. **(B,C)** Western blot analysis of PDLIM1 protein expression in IA and STA tissues. **(D)** GST pull-down assay further verifies the direct interaction between PDLIM1 and ACTN2. **(E,F)** Immunofluorescence staining illustrates the localization of PDLIM1 and ACTN2 proteins in IA and STA tissues. DAPI staining marks the nuclei, and merged images reveal the co-localization of the two proteins, highlighting their differential expression and potential interaction mechanisms in IA and STA tissues. **P < 0.01 vs. STA tissues.

**FIGURE 10:**
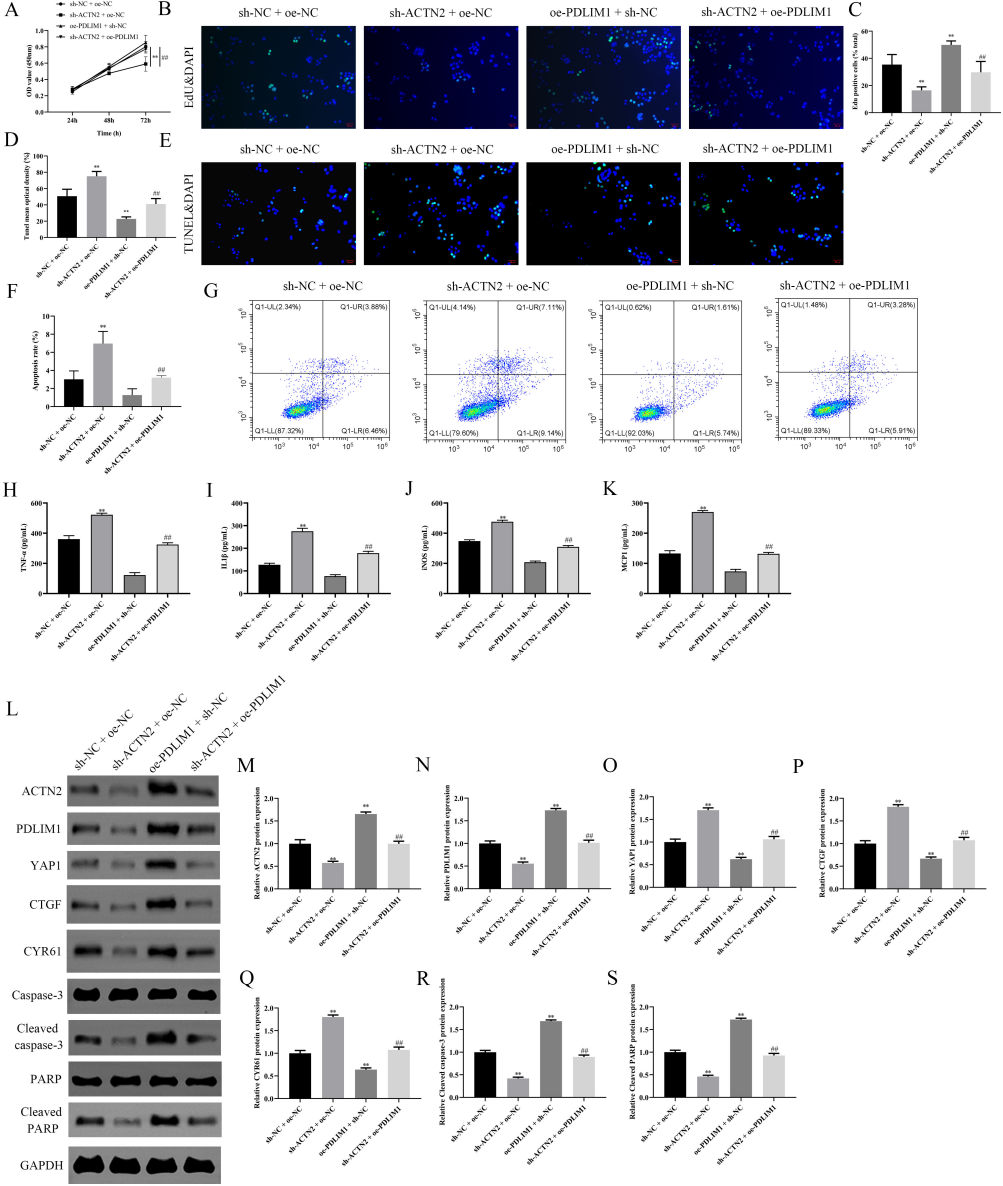
Effects of ACTN2 and PDLIM1 on Cell Proliferation, Apoptosis, Inflammatory Factors, and Related Protein Expression. **(A)** CCK8 assay measures cell viability at 24 h, 48 h, and 72 h in different treatment groups (sh-NC + oe-NC, sh-ACTN2 + oe-NC, oe-PDLIM1 + sh-NC, sh-ACTN2 + oe-PDLIM1). **(B,C)** EdU staining with DAPI nuclear staining demonstrates cell proliferation in each group, corroborating the above results. **(D,E)** TUNEL staining with DAPI nuclear staining illustrates cell apoptosis in each group. **(F,G)** Flow cytometry quantifies the apoptotic proportions in each group. **(H–K)** ELISA measures the expression levels of inflammatory factors TNF-α, IL-1β, iNOS, and MCP-1 in each group. **(L–S)** Western blot analysis of ACTN2, PDLIM1, YAP1, CTGF, CYR61, Caspase-3, Cleaved caspase-3, PARP, and Cleaved PARP protein expression in each group. **P < 0.01 vs. sh-NC + oe-NC, ##*P* < 0.01 vs. sh-ACTN2 + oe-NC.

We investigated the downstream targets of ACTN2 and their roles in VSMC function and aneurysm progression. Bioinformatics analysis identified PDLIM1 as a potential interactor of ACTN2. RT-PCR and WB confirmed significantly lower PDLIM1 expression in IA tissues compared to STA tissues (Figures 9A–C). Pull-down assays demonstrated a strong interaction between ACTN2 and PDLIM1 (Figure 9D), and FISH confirmed their co-localization in the cytoplasm (Figures 9E, F). Functional assays revealed that ACTN2 knockdown (sh-ACTN2) reduced VSMCs proliferation and increased apoptosis compared to controls. Overexpression of PDLIM1 (oe-PDLIM1) partially rescued these effects (Figures 10A–G). ELISA showed elevated levels of inflammatory cytokines (TNF-α, IL1β, iNOS, MCP1) in sh-ACTN2 cells, which were attenuated by PDLIM1 overexpression (Figures 10H–K). WB analysis indicated that ACTN2 knockdown suppressed the Hippo signaling pathway (YAP1, CTGF, CYR61) and increased apoptosis-related proteins (cleaved caspase-3, cleaved PARP), while PDLIM1 overexpression partially reversed these changes (Figures 10L–S).

### PDLIM1 promotes the proliferation of VSMCs and inhibits cell apoptosis and inflammation through Hippo-YAP1 signal pathway

**FIGURE 11:**
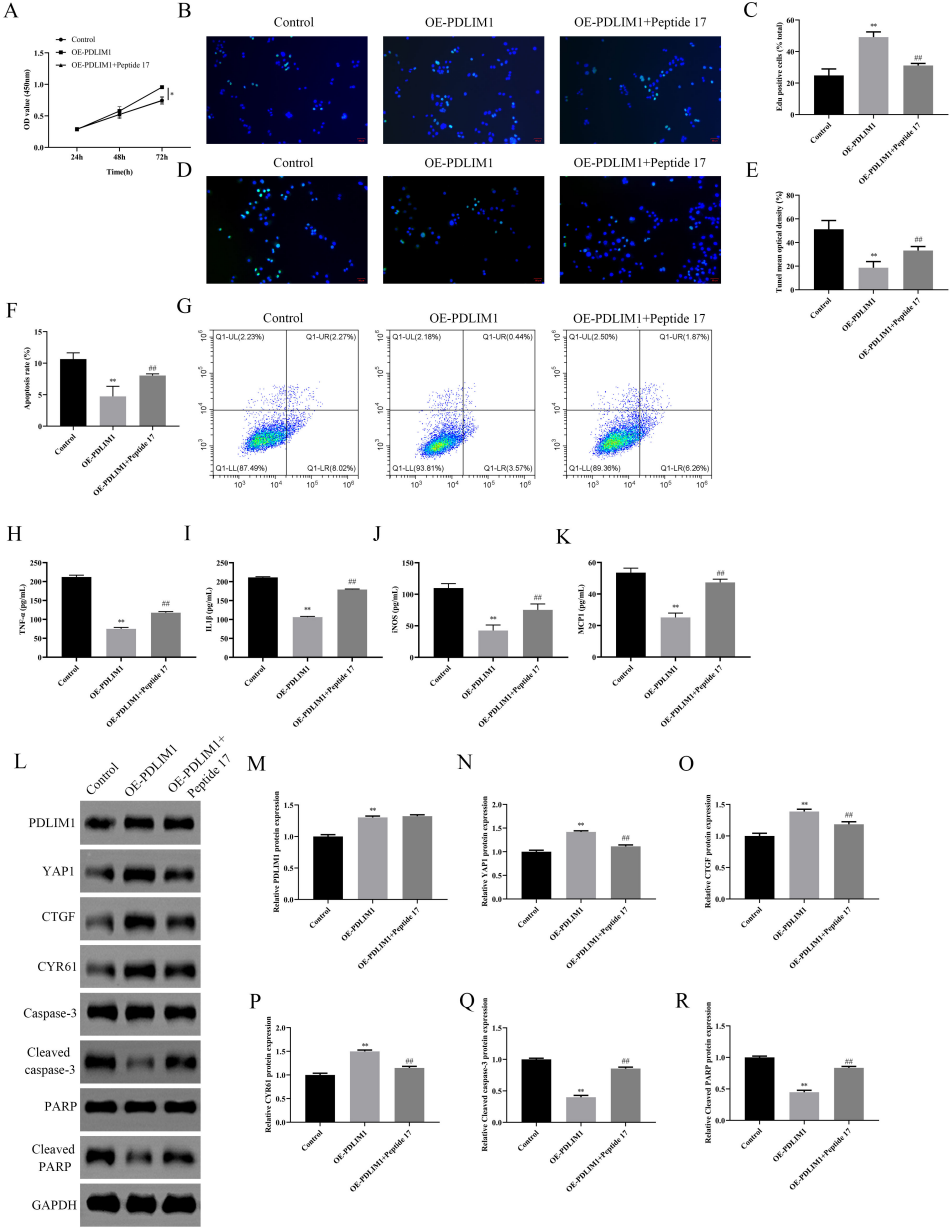
Effects of PDLIM1 Overexpression and Its Interaction with Peptide 17 on Cell Proliferation, Apoptosis, Inflammatory Factors, and Related Protein Expression. **(A)** CCK8 assay measures cell viability at 24 h, 48 h, and 72 h in different treatment groups (Control, OE-PDLIM1, OE-PDLIM1 + Peptide 17). **(B,C)** EdU staining with DAPI nuclear staining illustrates cell proliferation in each group. **(D,E)** TUNEL staining with DAPI nuclear staining demonstrates cell apoptosis in each group. **(F,G)** Flow cytometry quantifies the apoptotic proportions in each group. **(H–K)** Bar plots display the expression levels of inflammatory factors TNF-α, IL-1β, iNOS, and MCP-1 in each group. **(L–R)** Western blot analysis of PDLIM1, YAP1, CTGF, CYR61, Caspase-3, Cleaved caspase-3, PARP, and Cleaved PARP protein expression in each group. *P < 0.05 vs. Control, **P < 0.01 vs. Control, ##*P* < 0.01 vs. OE-PDLIM1.

We investigated the effects of PDLIM1 overexpression on VSMC proliferation, apoptosis, and related signaling pathways (Hippo signal pathway). CCK-8 and EdU assays demonstrated that overexpression of PDLIM1 significantly enhanced VSMC proliferation compared to the control group, as evidenced by increased OD values at 72 h (Figure 11A) and a higher number of EdU-positive cells (Figures 11B, C). However, the addition of Peptide 17 (the inhibitor of Hippo signal pathway) partially reversed these effects, indicating a potential interaction or inhibition mechanism (Figures 11A–C). TUNEL staining revealed that PDLIM1 overexpression reduced the apoptosis in VSMCs (Figures 11D, E), which was further confirmed by Flow cytometry analysis showing the lower rate of apoptosis (Figures 11F, G). The presence of Peptide 17 led to an increase in apoptosis, suggesting its role in modulating PDLIM1’s anti-apoptotic effects (Figures 11D–G). ELISA results indicated that PDLIM1 overexpression decreased the levels of inflammatory cytokines such as TNF-α, IL-1β, iNOS, and MCP-1, while Peptide 17 treatment restored these levels closer to those of the control group (Figures 11H–K). Western blot analysis showed that PDLIM1 overexpression upregulated Hippo pathway components YAP1, CTGF, and CYR61, and downregulated cleaved caspase-3 and cleaved PARP, markers of apoptosis (Figures 11L–R). These findings suggest that PDLIM1 plays a critical role in promoting VSMC proliferation, inhibiting apoptosis, and reducing inflammation, potentially through activation of the Hippo signaling pathway. The use of Peptide 17 provided insights into the specific mechanisms by which PDLIM1 exerts its effects, highlighting its therapeutic potential in vascular diseases.

### Knockdown of PDLIM1 can alleviate the progression of aneurysms in a mouse model

**FIGURE 12:**
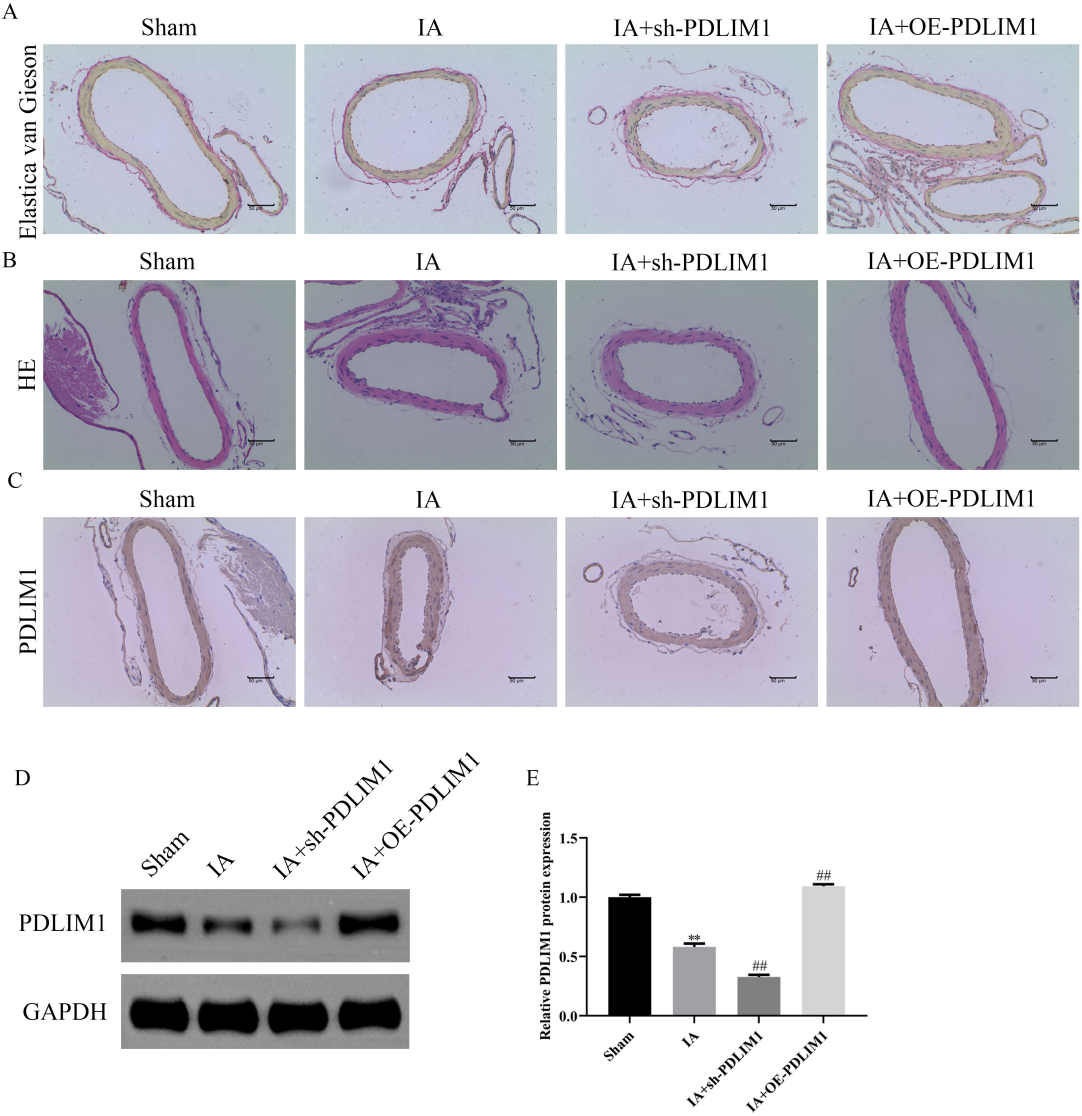
Expression of PDLIM1 in the IA Model and Its Impact on Vascular Structure. **(A)** Elastica van Gieson staining shows the distribution of elastic fibers in the vascular walls of different treatment groups (Sham, IA, IA + sh-PDLIM1, IA + OE-PDLIM1). **(B)** HE staining illustrates the morphological changes in vascular tissues. The Sham group exhibits intact vascular walls, the IA group shows significant thickening and inflammatory cell infiltration, the IA + OE-PDLIM1 group demonstrates improved vascular structure, and the IA + sh-PDLIM1 group exhibits more severe vascular damage. **(C)** Immunohistochemical staining detects PDLIM1 protein expression in vascular tissues of each group. **(D,E)** Western blot analysis of PDLIM1 protein expression in vascular tissues of each group. **P < 0.05 vs. Sham, **P < 0.05 vs. IA.

*In vivo*, Histological analysis via Elastica van Gieson and HE staining revealed severe vascular wall lesions in the aneurysm model and sh-PDLIM1 groups, characterized by collagen layer loss, fibrosis, and VSMC thinning, whereas oe-PDLIM1 resulted in milder lesions (Figures 12A, B). Immunohistochemistry and WB confirmed reduced PDLIM1 expression in the aneurysm model and sh-PDLIM1 groups compared to controls, with oe-PDLIM1 restoring expression (Figures 12C–E). These findings suggest that PDLIM1 acts as a downstream effector of ACTN2, regulating VSMC proliferation, apoptosis, and inflammation, and plays a critical role in aneurysm progression, highlighting its potential as a therapeutic target.

## Discussion

Intracranial aneurysm (IA), characterized by abnormal dilation of blood vessels, represent a significant clinical challenge due to their potential for rupture and life-threatening complications (Tang et al., 2019). Recent advances in vascular biology have highlighted the pivotal role of cytoskeletal proteins in maintaining vascular integrity and homeostasis (Rui et al., 2017). Proteins such as MYH11 and ACTA2, known for their involvement in smooth muscle contraction and structural stability, have been implicated in aneurysm pathogenesis through their influence on vascular smooth muscle cell (VSMC) function (Burger et al., 2021). These findings underscore the importance of the cytoskeleton in vascular remodeling and disease progression. This study establishes ACTN2 as a critical regulator in arterial aneurysm progression, bridging cytoskeletal dynamics and vascular remodeling. Through bioinformatics analysis and experimental validation, we identified significant downregulation of ACTN2 in aneurysmal tissues, correlating with aberrant VSMC proliferation and suppressed apoptosis. Moreover, our work uniquely implicates ACTN2 in modulating synaptic pathway-related genes, suggesting a novel neurovascular regulatory axis. The functional rescue of ACTN2 in VSMCs further underscores its therapeutic potential, offering a molecular target to counteract pathological vascular remodeling.

In recent years, PRDM9, as a histone lysine methyltransferase, has garnered significant attention due to its pivotal role in epigenetic regulation (Di Tullio et al., 2022). PRDM9 catalyzes the trimethylation of lysine 4 on histone H3 (H3K4me3), thereby participating in gene transcription activation and chromatin remodeling (Di Tullio et al., 2022). In our study, the USUC database predicted a substantial occurrence of H3K4me3 modifications near the ACTN2 promoter, and CHIP-qPCR and immunohistochemistry revealed that low levels of H3K4me3 modifications drive the suppression of ACTN2. Additionally, the USUC database predicted the presence of PRDM9 near the ACTN2 promoter, and CHIP-qPCR and co-localization experiments confirmed PRDM9’s involvement in the transcriptional regulation of ACTN2. We hypothesize that low expression of PRDM9 inhibits H3K4me3, thereby reducing ACTN2 expression and subsequently affecting the progression of aneurysms. In exploring the relationship between single nucleotide polymorphisms (SNPs) and IA in the Kazakh population, linear regression analysis identified 13 SNPs significantly associated with the occurrence and rupture of IA, including rs3932338 located 214 kilobases downstream of PRDM9 (Zholdybayeva et al., 2018). Glutamine enhances the recruitment of C/EBPb to the PRDM9 enhancer region, regulating the gene transcription of PRDM9 (Pan et al., 2023). The activation of PRDM9 further drives H3K4me3 modifications, promoting the transcriptional reprogramming of genes related to adipogenesis and thermogenesis (Pan et al., 2023). In this study, the depletion of super enhancers (SE) indeed led to the downregulation of ACTN2 expression in VSMCs, further proving that the low expression of ACTN2 is dependent on the SE regulation by PRDM9. Subsequently, the overexpression plasmid of PRDM9 successfully eliminated the effects of ACTN2 knockdown on VSMC function *in vitro*, once again validating our theory.

Bioinformatics analysis revealed that PDLIM1 is one of the targets of ACTN2. PDLIM1, as a scaffold protein crucial for mechanotransduction (Ye et al., 2023), promotes the nuclear translocation of YAP/TAZ in the absence of ACTN2. This mechanism aligns with the dysregulation of the Hippo pathway observed in previous studies on aortic aneurysms (Yan et al., 2024). PDLIM1 has been reported to be downregulated in endothelial cells of IA, and silencing PDLIM1 inhibits the viability, migration, and tube-forming ability of vascular endothelial cells by modulating the activity of the Wnt/β-catenin signaling pathway (Yan et al., 2024). Several studies have reported that PDLIM1 is involved in regulating the Hippo pathway (Huang et al., 2020; Tan et al., 2022; Huang et al., 2024). Yes-associated protein (YAP), as a transcriptional co-activator, is a downstream effector of the Hippo pathway (Tan et al., 2022). Increasing evidence suggests that the Hippo/YAP pathway plays a significant role in vascular remodeling and related cardiovascular diseases (Zhou and Zhao, 2018; Zheng et al., 2022; Wu et al., 2024). The Hippo/YAP pathway participates in vascular remodeling processes in cardiovascular diseases such as pulmonary hypertension, atherosclerosis, restenosis, aortic aneurysms, and angiogenesis by altering the production or degradation of the extracellular matrix and regulating the growth, death, and migration of vascular smooth muscle cells (VSMCs) and endothelial cells (Yu et al., 2020; Pulkkinen et al., 2021; Quan et al., 2022; Liu et al., 2023; Kong et al., 2024). In patients with Stanford type A aortic dissection, YAP expression in the ascending aortic wall is significantly reduced (Li et al., 2016). This phenomenon was further validated in a mouse model of Stanford type A aortic dissection induced by BAPN (Jiang et al., 2016). Studies have shown that the absence of YAP significantly promotes VSMC apoptosis under static conditions *in vitro*, while changes in mechanical stress directly induce downregulation of YAP expression and exacerbate VSMC apoptosis (Jiang et al., 2016). Research has reported that NETs induce a synthetic and pro-inflammatory smooth muscle cell (SMC) phenotype in a Hippo-YAP pathway-dependent manner, promoting the formation of abdominal aortic aneurysms (Yang et al., 2023). In our study, RNA pull-down and RIP experiments confirmed that ACTN2 binds to PDLIM1 mRNA and enhances its stability. Further functional assays demonstrated that overexpression of PDLIM1 can reverse the effects of ACTN2 knockdown on VSMCs. Moreover, inhibition of the Hippo-YAP pathway can reverse the effects of PDLIM1 overexpression on VSMCs. Therefore, the ACTN2-PDLIM1-Hippo axis may serve as a central hub in the progression of aneurysms.

Despite these advancements, several limitations need to be addressed. Firstly, the clinical relevance of ACTN2 in different aneurysm subtypes (such as intracranial aneurysms and aortic aneurysms) requires further validation in large-scale cohorts. Secondly, the interactions between ACTN2 and other cytoskeletal regulatory factors (such as MYL9 and CFL2) warrant exploration to clarify compensatory regulatory networks. Future studies should employ single-cell sequencing technologies to dissect the cell type-specific functions of ACTN2 and develop genetically engineered models (e.g., VSMC-specific ACTN2/PRDM9 knockout) to optimize therapeutic strategies.

## Conclusion

In summary, our study demonstrates that ACTN2 is downregulated in IA tissues. Furthermore, this research elucidates that PRDM9-induced ACTN2 promotes the proliferation and inhibits the apoptosis and inflammation of VSMCs in IA through the PDLIM1-Hippo axis. The discovery of this axis and its impact on IA will contribute to advancing IA research and exploring effective therapeutic strategies for IA.

## Data Availability

The protein-protein interaction data used in this study were obtained from the STRING database (https://string-db.org/). All data were processed and presented in accordance with the terms of use and citation guidelines of the STRING database. Further enquires should be directed to the corresponding author.

## Footnotes

1
https://www.ncbi.nlm.nih.gov/geo/

## References

[B1] Baker C. Walker M. Kajita S. Petkov P. Paigen K. (2014). PRDM9 binding organizes hotspot nucleosomes and limits Holliday junction migration. *Genome Res.* 24 724–732. 10.1101/gr.170167.113 24604780 PMC4009602

[B2] Bauer K. Kratzer M. Otte M. de Quintana K. Hagmann J. Arnold G. (2000). Human CLP36, a PDZ-domain and LIM-domain protein, binds to alpha-actinin-1 and associates with actin filaments and stress fibers in activated platelets and endothelial cells. *Blood* 96 4236–4245. 10.1182/blood.V96.13.4236 11110697

[B3] Burger J. Bogunovic N. de Wagenaar N. Liu H. van Vliet N. IJpma A. (2021). Molecular phenotyping and functional assessment of smooth muscle-like cells with pathogenic variants in aneurysm genes ACTA2, MYH11, SMAD3 and FBN1. *Hum. Mol. Genet.* 30 2286–2299. 10.1093/hmg/ddab190 34244757 PMC8600030

[B4] Chen C. Chang S. Lin C. Chern S. Wu P. Chen S. (2018). Prenatal diagnosis of a familial 5p14.3-p14.1 deletion encompassing CDH18, CDH12, PMCHL1, PRDM9 and CDH10 in a fetus with congenital heart disease on prenatal ultrasound. *Taiwan J. Obstet Gynecol.* 57 734–738. 10.1016/j.tjog.2018.08.023 30342662

[B5] ChenQ.LiuS.ZhouH.WangJ.XiaoX.ChenG. (2025). SAMD4A inhibits abdominal aortic aneurysm development and VSMC phenotypic transformation through targeting KDM2B. *J. Adv. Res.* [Online ahead of print]. 10.1016/j.jare.2025.03.018 40081568

[B6] Di Tullio F. Schwarz M. Zorgati H. Mzoughi S. Guccione E. (2022). The duality of PRDM proteins: Epigenetic and structural perspectives. *FEBS J.* 289 1256–1275. 10.1111/febs.15844 33774927 PMC8979648

[B7] Etminan N. Dörfler A. Steinmetz H. (2020). Unruptured intracranial aneurysms- pathogenesis and individualized management. *Deutsches Arzteblatt Int.* 117 235–242. 10.3238/arztebl.2020.0235 32449895 PMC7264289

[B8] Etminan N. Rinkel G. (2016). Unruptured intracranial aneurysms: Development, rupture and preventive management. *Nat. Rev. Neurol.* 12 699–713. 10.1038/nrneurol.2016.150 27808265

[B9] Hallikainen J. Keränen S. Savolainen J. Närhi M. Suominen A. Ylöstalo P. (2021). Role of oral pathogens in the pathogenesis of intracranial aneurysm: Review of existing evidence and potential mechanisms. *Neurosurg. Rev.* 44 239–247. 10.1007/s10143-020-01253-y 32034564 PMC7850994

[B10] Hu Y. Chen W. Li C. Wang X. Luo J. Cheng B. (2022). LncRNA ANRIL facilitates vascular smooth muscle cell proliferation and suppresses apoptosis via modulation of miR-7/FGF2 pathway in intracranial aneurysms. *Neurocrit. Care* 36 106–115. 10.1007/s12028-021-01262-9 34286462

[B11] Huang Q. Huang Q. Sun Y. Wu S. (2019). High-throughput data reveals novel circular RNAs via competitive endogenous RNA networks associated with human intracranial aneurysms. *Med. Sci. Monit.* 25 4819–4830. 10.12659/MSM.917081 31254341 PMC6615076

[B12] Huang X. Zhou L. Feng W. Liu Y. Chen M. Tang L. (2024). Circ ubiquitin-like-containing plant homeodomain and RING finger domains protein 1 increases the stability of G9a and ubiquitin-like-containing plant homeodomain and RING finger domains protein 1 messenger RNA through recruiting eukaryotic translation initiation factor 4A3, transcriptionally inhibiting PDZ and homeobox protein domain protein 1, and promotes the metastasis of hepatocellular carcinoma. *J. Gastroenterol. Hepatol.* 39 596–607. 10.1111/jgh.16408 38059880

[B13] Huang Z. Zhou J. Wang K. Chen H. Qin S. Liu J. (2020). PDLIM1 inhibits tumor metastasis through activating hippo signaling in hepatocellular carcinoma. *Hepatology* 71 1643–1659. 10.1002/hep.30930 31509262

[B14] Jiang W. Ren W. Liu X. Liu Y. Wu F. Sun L. (2016). Disruption of mechanical stress in extracellular matrix is related to Stanford type A aortic dissection through down-regulation of Yes-associated protein. *Aging* 8 1923–1939. 10.18632/aging.101033 27608489 PMC5076445

[B15] Jie H. Wang B. Zhang J. Wang X. Song X. Yang F. (2024). Uncovering SPP1(+) macrophage, neutrophils and their related diagnostic biomarkers in intracranial aneurysm and subarachnoid hemorrhage. *J. Inflamm. Res.* 17 8569–8587. 10.2147/JIR.S493828 39539729 PMC11559423

[B16] Kong D. Liu J. Lu J. Zeng C. Chen H. Duan Z. (2024). HMGB2 release promotes pulmonary hypertension and predicts severity and mortality of patients with pulmonary arterial hypertension. *Arterioscler. Thromb. Vasc. Biol.* 44 e172–e195. 10.1161/ATVBAHA.123.319916 38572649

[B17] Li H. Jiang W. Ren W. Guo D. Guo J. Wang X. (2016). Downregulation of the yes-associated protein is associated with extracellular matrix disorders in ascending aortic aneurysms. *Stem Cells Int.* 2016:6786184. 10.1155/2016/6786184 26904131 PMC4745276

[B18] Lindholm M. Jimenez-Morales D. Zhu H. Seo K. Amar D. Zhao C. (2021). Mono- and biallelic protein-truncating variants in alpha-actinin 2 cause cardiomyopathy through distinct mechanisms. *Circ. Genomic Precis. Med.* 14:e003419. 10.1161/CIRCGEN.121.003419 34802252 PMC8692448

[B19] Liu H. Sun M. Wu N. Liu B. Liu Q. Fan X. (2023). TGF-β/Smads signaling pathway, Hippo-YAP/TAZ signaling pathway, and VEGF: Their mechanisms and roles in vascular remodeling related diseases. *Immun. Inflamm. Dis.* 11:e1060. 10.1002/iid3.1060 38018603 PMC10629241

[B20] Maimaiti A. Turhon M. Abulaiti A. Dilixiati Y. Zhang F. Axieer A. (2023). DNA methylation regulator-mediated modification patterns and risk of intracranial aneurysm: A multi-omics and epigenome-wide association study integrating machine learning, Mendelian randomization, eQTL and mQTL data. *J. Transl. Med.* 21:660. 10.1186/s12967-023-04512-w 37742034 PMC10518114

[B21] Miyata T. Minami M. Kataoka H. Hayashi K. Ikedo T. Yang T. (2020). Osteoprotegerin prevents intracranial aneurysm progression by promoting collagen biosynthesis and vascular smooth muscle cell proliferation. *J. Am. Heart Assoc.* 9:e015731. 10.1161/JAHA.119.015731 32856519 PMC7660769

[B22] Pan W. Gao Y. Wan W. Xiao W. You C. (2021). LncRNA SAMMSON overexpression suppresses vascular smooth muscle cell proliferation via inhibiting miR-130a maturation to participate in intracranial aneurysm. *Neuropsychiatr. Dis. Treat.* 17 1793–1799. 10.2147/NDT.S311499 34113109 PMC8187098

[B23] Pan X. Ye L. Guo X. Wang W. Zhang Z. Wang Q. (2023). Glutamine production by glul promotes thermogenic adipocyte differentiation through prdm9-mediated H3K4me3 and transcriptional reprogramming. *Diabetes* 72 1574–1596. 10.2337/db23-0162 37579296

[B24] Pontes F. da Silva E. Baptista-Silva J. Vasconcelos V. (2019). Treatments for unruptured intracranial aneurysms. *Cochrane Database Syst. Rev.* 5:CD013312. 10.1002/14651858.CD013312 PMC8109849 33971026

[B25] Pulkkinen H. Kiema M. Lappalainen J. Toropainen A. Beter M. Tirronen A. (2021). BMP6/TAZ-Hippo signaling modulates angiogenesis and endothelial cell response to VEGF. *Angiogenesis* 24 129–144. 10.1007/s10456-020-09748-4 33021694 PMC7921060

[B26] Quan M. Lv H. Liu Z. Li K. Zhang C. Shi L. (2022). MST1 suppresses disturbed flow induced atherosclerosis. *Circ. Res.* 131 748–764. 10.1161/CIRCRESAHA.122.321322 36164986

[B27] Rui Y. Xu Z. Fang X. Menezes M. Balzeau J. Niu A. (2017). The intracranial aneurysm gene THSD1 connects endosome dynamics to nascent focal adhesion assembly. *Cell Physiol. Biochem.* 43 2200–2211. 10.1159/000484298 29069646

[B28] Tan Y. Li Y. Zhu H. Wu X. Mei K. Li P. (2022). miR-187/PDLIM1 gets involved in gastric cancer progression and cisplatin sensitivity of cisplatin by mediating the Hippo-YAP signaling pathway. *J. Oncol.* 2022:5456016. 10.1155/2022/5456016 36164345 PMC9509220

[B29] Tang H. Luo Y. Zuo Q. Wang C. Huang Q. Zhao R. (2019). Current understanding of the molecular mechanism between hemodynamic- induced intracranial aneurysm and inflammation. *Curr. Protein Pept. Sci.* 20 789–798. 10.2174/1389203720666190507101506 31060483

[B30] Wang K. Wang Y. Wan H. Wang J. Hu L. Huang S. (2024). Actn2 defects accelerates H9c2 hypertrophy via ERK phosphorylation under chronic stress. *Genes Genomics* 46 1013–1022. 10.1007/s13258-024-01536-4 38990270

[B31] Wang Z. Ma J. Yue H. Zhang Z. Fang F. Wang G. (2023). Vascular smooth muscle cells in intracranial aneurysms. *Microvasc. Res.* 149:104554. 10.1016/j.mvr.2023.104554 37236346

[B32] Wu H. Che Y. Lan Q. He Y. Liu P. Chen M. (2024). The multifaceted roles of hippo-YAP in cardiovascular diseases. *Cardiovasc. Toxicol.* 24 1410–1427. 10.1007/s12012-024-09926-6 39365552

[B33] Yan Y. Qin X. Zheng Y. Jin T. Hu Y. An Q. (2024). Decreased PDLIM1 expression in endothelial cells contributes to the development of intracranial aneurysm. *Vasc. Med.* 29 5–16. 10.1177/1358863X231218210 38334094

[B34] Yang S. Chen L. Wang Z. Chen J. Ni Q. Guo X. (2023). Neutrophil extracellular traps induce abdominal aortic aneurysm formation by promoting the synthetic and proinflammatory smooth muscle cell phenotype via Hippo-YAP pathway. *Transl. Res.* 255 85–96. 10.1016/j.trsl.2022.11.010 36435329

[B35] Yao Y. Huang J. Geng Y. Qian H. Wang F. Liu X. (2015). Paracrine action of mesenchymal stem cells revealed by single cell gene profiling in infarcted murine hearts. *PLoS One* 10:e0129164. 10.1371/journal.pone.0129164 26043119 PMC4456391

[B36] Ye B. Yu M. Yue M. Yin M. Zhang C. Wang Q. (2023). Role of PDLIM1 in hepatic stellate cell activation and liver fibrosis progression. *Sci. Rep.* 13:10946. 10.1038/s41598-023-38144-3 37414929 PMC10326060

[B37] Yu Y. Su X. Qin Q. Hou Y. Zhang X. Zhang H. (2020). Yes-associated protein and transcriptional coactivator with PDZ-binding motif as new targets in cardiovascular diseases. *Pharmacol. Res.* 159:105009. 10.1016/j.phrs.2020.105009 32553712

[B38] Zhang Z. Mu X. Zhou X. (2022). Dexmedetomidine alleviates inflammatory response and oxidative stress injury of vascular smooth muscle cell via α2AR/GSK-3β/MKP-1/NRF2 axis in intracranial aneurysm. *BMC Pharmacol. Toxicol.* 23:81. 10.1186/s40360-022-00607-0 36273189 PMC9588221

[B39] Zheng A. Chen Q. Zhang L. (2022). The Hippo-YAP pathway in various cardiovascular diseases: Focusing on the inflammatory response. *Front. Immunol* 13:971416. 10.3389/fimmu.2022.971416 36059522 PMC9433876

[B40] Zholdybayeva E. Medetov Y. Aitkulova A. Makhambetov Y. Akshulakov S. Kaliyev A. (2018). Genetic risk factors for intracranial aneurysm in the kazakh population. *J. Mol. Neurosci.* 66 135–145. 10.1007/s12031-018-1134-y 30121816

[B41] Zhou W. Zhao M. (2018). How hippo signaling pathway modulates cardiovascular development and diseases. *J. Immunol. Res.* 2018:3696914. 10.1155/2018/3696914 29577047 PMC5822808

